# Regulation of mRNA Levels by Decay-Promoting Introns that Recruit the Exosome Specificity Factor Mmi1

**DOI:** 10.1016/j.celrep.2015.11.026

**Published:** 2015-12-06

**Authors:** Cornelia Kilchert, Sina Wittmann, Monica Passoni, Sneha Shah, Sander Granneman, Lidia Vasiljeva

**Affiliations:** 1 Department of Biochemistry, University of Oxford, South Parks Road, Oxford OX1 3QU, UK; 2 Institute for Structural and Molecular Biology, Centre for Synthetic and Systems Biology (SynthSys), C.H. Waddington Building, the King’s Buildings, Mayfield Road, Edinburgh EH9 3BF, UK

**Keywords:** splicing, mRNA, Mmi1, RNA exosome, RNA decay, intron retention

## Abstract

In eukaryotic cells, inefficient splicing is surprisingly common and leads to the degradation of transcripts with retained introns. How pre-mRNAs are committed to nuclear decay is unknown. Here, we uncover a mechanism by which specific intron-containing transcripts are targeted for nuclear degradation in fission yeast. Sequence elements within these “decay-promoting” introns co-transcriptionally recruit the exosome specificity factor Mmi1, which induces degradation of the unspliced precursor and leads to a reduction in the levels of the spliced mRNA. This mechanism negatively regulates levels of the RNA helicase DDX5/Dbp2 to promote cell survival in response to stress. In contrast, fast removal of decay-promoting introns by co-transcriptional splicing precludes Mmi1 recruitment and relieves negative expression regulation. We propose that decay-promoting introns facilitate the regulation of gene expression. Based on the identification of multiple additional Mmi1 targets, including mRNAs, long non-coding RNAs, and sn/snoRNAs, we suggest a general role in RNA regulation for Mmi1 through transcript degradation.

## Highlights

• “Decay-promoting” introns contain sequences that activate nuclear mRNA decay
• Fast splicing of decay-promoting introns prevents recruitment of decay factors
• In contrast, increased intron retention under stress results in low gene expression
• This mechanism acts to promote cell survival in response to stress

## Graphical Abstract

**Figure:**
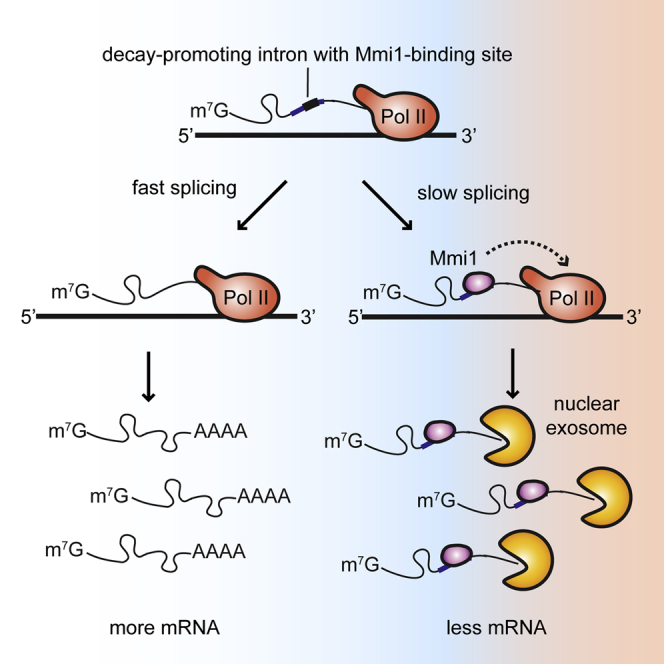

## Teaser

Inefficient splicing is common and leads to the degradation of transcripts with retained introns. Kilchert et al. show that “decay-promoting” introns harbor sites that recruit RNA decay factors. Although fast splicing precludes recruitment of the nuclear surveillance machinery, increased intron retention reduces gene expression, which can promote cell survival under stress.

## Introduction

To produce functional mRNAs, introns in nascent pre-mRNAs must be removed by splicing. Recent studies suggest that splicing is a highly regulated process and responsible for changes in gene expression during differentiation or in response to environmental cues (Bergkessel et al., 2011, Braunschweig et al., 2014, Colak et al., 2013, Parenteau et al., 2011, Pleiss et al., 2007, Wong et al., 2013, Yap et al., 2012). In higher eukaryotes in particular, the use of alternative splice sites increases protein diversity by yielding variant proteins. In addition, regulated splicing can also impact mRNA levels. For example, intron retention (IR) can lead to the degradation of pre-mRNAs in the cytoplasm by nonsense-mediated mRNA decay (NMD) (Wong et al., 2013). In yeast, IR is widespread and results in the degradation of pre-mRNAs by the nuclear exosome complex (Bousquet-Antonelli et al., 2000, Gudipati et al., 2012, Lemieux et al., 2011, Schneider et al., 2012), but how pre-mRNAs are committed to decay by the exosome remains poorly understood.

The exosome is a conserved multi-subunit ribonuclease with various functions in RNA metabolism (reviewed in Chlebowski et al., 2013). It consists of a non-catalytic core and two associated 3′ to 5′ exonucleases, Rrp6 and Dis3. The exosome relies on co-factors for optimal activity. One of the best studied is the *Saccharomyces cerevisiae* Trf4/5-Air1/2-Mtr4 polyadenylation (TRAMP) complex consisting of the RNA helicase Mtr4, the polyA (pA) polymerase Trf4, and the RNA-binding protein Air1 or Air2. TRAMP adds short pA tails to transcripts (reviewed in Schmidt and Butler, 2013). In contrast to their stabilizing function in the cytoplasm, nuclear pA tails have been linked to RNA degradation and nuclear pA-binding proteins to downregulation of pre-mRNA levels in both yeast and mammals (Bergeron et al., 2015, Lemieux et al., 2011, Muniz et al., 2015, Schmid et al., 2012). In the fission yeast *Schizosaccharomyces pombe*, the Trf4 homolog Cid14 appears to play a less prominent role in RNA decay. Instead, the Mtr4-like helicase Mtl1 and the RNA-binding protein Red1 (Mtl1-Red1 core, MTREC) associate with the canonical pA polymerase Pla1 and function as central activators of the nuclear exosome (Egan et al., 2014, Lee et al., 2013, Zhou et al., 2015). The nuclear pA-binding protein Pab2 also associates with MTREC. The precise mechanisms through which MTREC is targeted to its broad range of specific substrates remains unknown.

Approximately 30 meiotic mRNAs are known to be unstable during mitotic growth because they carry “determinants of selective removal” (DSRs), RNA sequences that are enriched for the hexanucleotide motif U(U/C/G)AAAC (Chen et al., 2011, Harigaya et al., 2006, Yamashita et al., 2012). This results in co-transcriptional recruitment of the conserved RNA-binding protein Mmi1 to target genes, which programs transcripts for decay by the exosome (Chen et al., 2011, Harigaya et al., 2006, Hiriart et al., 2012, Tashiro et al., 2013, Yamashita et al., 2012, Zofall et al., 2012). In addition, Mmi1 has been reported to regulate RNA processing, including splicing and 3′ end formation (Chen et al., 2011, McPheeters et al., 2009, Shah et al., 2014). Mmi1-dependent RNA decay has been linked to the presence of pA tails on targeted transcripts, which are frequently extended (St-André et al., 2010, Yamanaka et al., 2010, Zhou et al., 2015). This Mmi1-dependent hyperadenylation is executed by Pla1 rather than by the TRAMP complex (Chen et al., 2011, Yamanaka et al., 2010). Moreover, degradation of meiotic transcripts also requires Pab2 (St-André et al., 2010). Recent studies have demonstrated that Mmi1 co-purifies with MTREC subunits (Egan et al., 2014, Lee et al., 2013, Sugiyama and Sugioka-Sugiyama, 2011), suggesting that Mmi1 could provide specificity to MTREC and act more broadly in gene regulation. We and others have recently found Mmi1 to also induce turnover of non-coding RNAs (ncRNAs) that regulate phosphate-responsive genes (Ard et al., 2014, Shah et al., 2014), supporting the idea that Mmi1 does not exclusively target meiotic transcripts. Previous studies aimed at the identification of Mmi1 targets relied on microarray analyses in temperature-sensitive *mmi1* mutants (Chen et al., 2011, Harigaya et al., 2006). Although these experiments provided valuable insight into gene networks regulated by Mmi1, they failed to distinguish between direct and indirect targets.

Here, we perform UV cross-linking and analysis of cDNA (CRAC) to identify Mmi1 RNA substrates. Unexpectedly, our results demonstrate that Mmi1 associates with RNAs synthesized by all three RNA polymerases (Pol), I, II, and III. To determine how Mmi1 contributes to the regulation of RNA metabolism, we used RNA sequencing (RNA-seq) to measure changes in RNA expression levels in a *mmi1* deletion strain (*mmi1Δ*). We report that expression of many protein-coding and ncRNAs is significantly changed in *mmi1Δ*, suggesting that Mmi1 could act as a global regulator of mRNA metabolism. However, based on our data, we conclude that the main role of Mmi1 is the activation of RNA decay. Substrates include pre-mRNAs, ncRNAs, and 3′-extended RNAs that are products of leaky transcription termination.

Importantly, we show that, in some cases, Mmi1 preferably binds introns, suggesting that Mmi1 function on these transcripts depends on splicing. By studying the regulation of two individual examples, *dbp2* and *rps2202*, which encode a conserved essential RNA helicase and the ribosomal protein S15a, respectively, we show that Mmi1 binding to the intron reduces gene expression levels under conditions that favor IR. If conditions allow fast splicing, then Mmi1 is no longer recruited, leading to increased levels of mRNA. Strikingly, deletion of the introns deregulates gene expression and alters cell viability in response to environmental stresses. We propose that these “decay-promoting” introns facilitate the regulation of gene expression in response to environmental cues.

## Results

### Identification of Mmi1 RNA Targets and the Binding Motif

**Figure 1:**
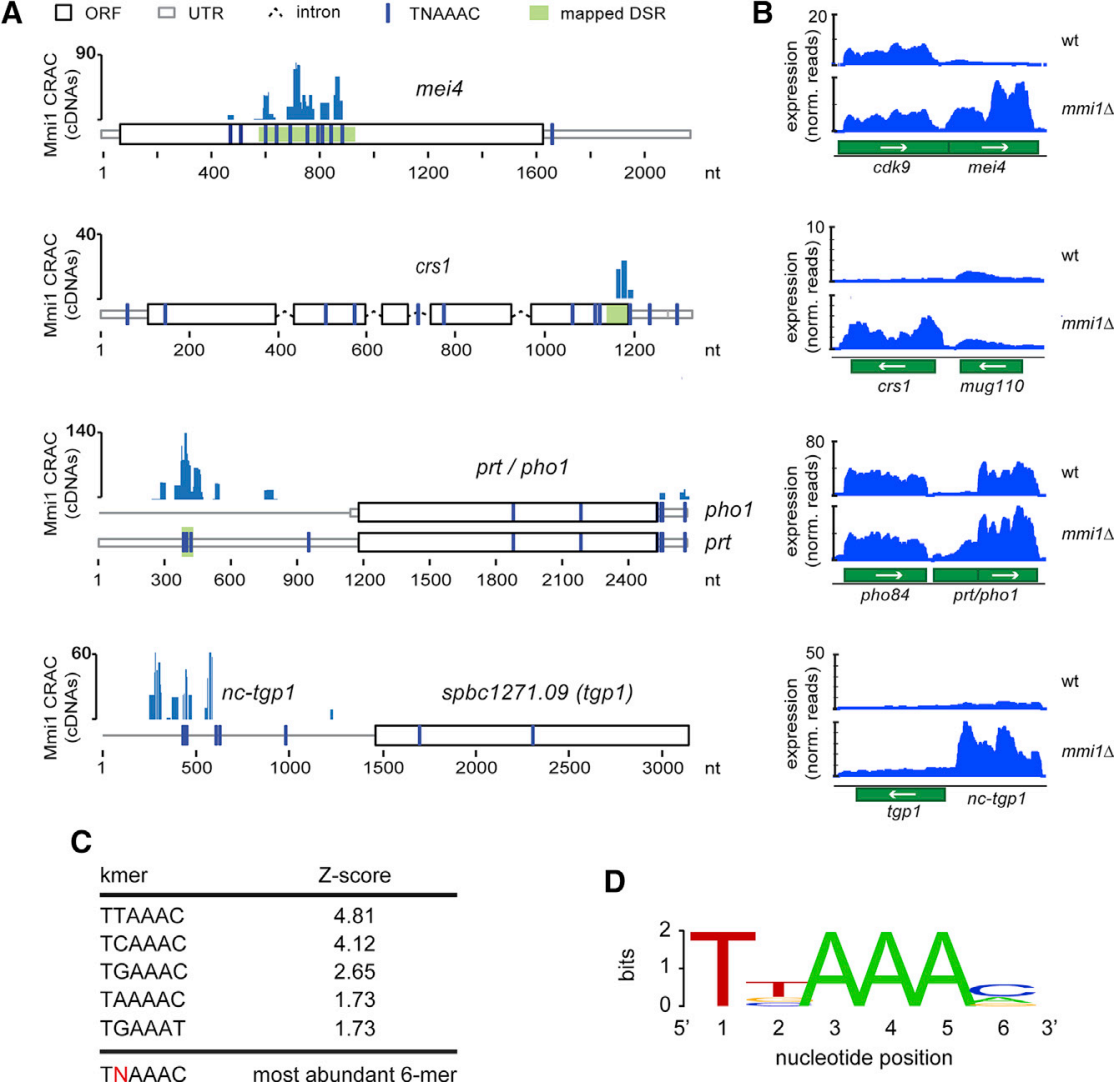
Identification of Mmi1 RNA Targets and the Binding Motif (A) Mmi1 CRAC cDNA read distribution over four well described Mmi1 targets, the meiotic mRNAs *mei4* and *crs1* and ncRNAs *prt/pho1* and *nc-tgp1*. The positions of the TNAAAC motifs are indicated. Previously mapped DSRs are indicated in green (Harigaya et al., 2006, McPheeters et al., 2009, Shah et al., 2014). ORF, open reading frame; nt, nucleotide. (B) RNA-seq analysis of the Mmi1 target genes shown in (A). norm., normalized. (C) Hexamers that closely resemble the consensus Mmi1 motif were enriched significantly in the CRAC data. (D) Mmi1-binding motifs enriched in cDNA clusters that mapped to mRNAs. Motif analysis was performed using the pyMotif tool in the pyCRAC package. See also Figure S1.

To identify direct Mmi1 RNA substrates, we performed CRAC. Actively growing cells were UV-irradiated using the Megatron, and cross-linked Mmi1 substrates were purified by stringent two-step affinity purification and sequenced (Granneman et al., 2009, Granneman et al., 2011). Untagged strains served as negative controls. We detected efficient cross-linking with previously described targets of Mmi1, such as the meiotic mRNAs *mei4* and *crs1* or the regulatory phosphate-responsive ncRNAs *prt* and *nc-tgp1* (Ard et al., 2014, Harigaya et al., 2006, McPheeters et al., 2009, Shah et al., 2014; Figure 1A). To complement these analyses, we performed RNA-seq to measure changes in gene expression in an *mmi1Δ* strain. RNA-seq analyses were performed in triplicate, and differential expression was analyzed using DESeq2 (Love et al., 2014). Consistent with previous work, our RNA-seq data analyses revealed a significant accumulation of known Mmi1 targets in the *mmi1Δ* strain (Figure 1B). 30 non-overlapping transcripts had been assigned to the “Mmi1 regulon” based on their altered expression in *mmi1-ts* mutants (Chen et al., 2011). Of these, 19 cross-linked to Mmi1. For three of the regulon genes, CRAC reads mapped anti-sense to the gene (Figure S1A; Table S1). To identify the Mmi1-binding motif, read clusters were subjected to K-mer analyses using pyMotif from the pyCRAC package (Webb et al., 2014). In agreement with the previously defined Mmi1-binding motif (Chen et al., 2011, Yamashita et al., 2012), TNAAAC hexamers were enriched significantly (Figures 1C and 1D). The position of Mmi1 cross-links along transcripts appeared to be random (Figure S1B).

Surprisingly, our analysis revealed Mmi1 binding to diverse classes of RNAs produced by Pol I, II, and III. In addition to ∼450 protein-coding and ncRNA genes transcribed by Pol II, Mmi1 cross-linked to small nuclear RNAs (snRNAs)/small nucleolar RNAs (snoRNAs) and many Pol III transcripts. The long ribosomal precursor generated by Pol I contributed strongly to the total number of reads. 13.5% of total reads mapped to intergenic regions, consistent with a role for Mmi1 in regulating long non-coding RNA (lncRNA) expression and/or quality control of 3′ end processing of RNAs.

### Low-Abundance Transcripts Are Highly Enriched in Mmi1 CRAC Sequencing Data

**Figure 2:**
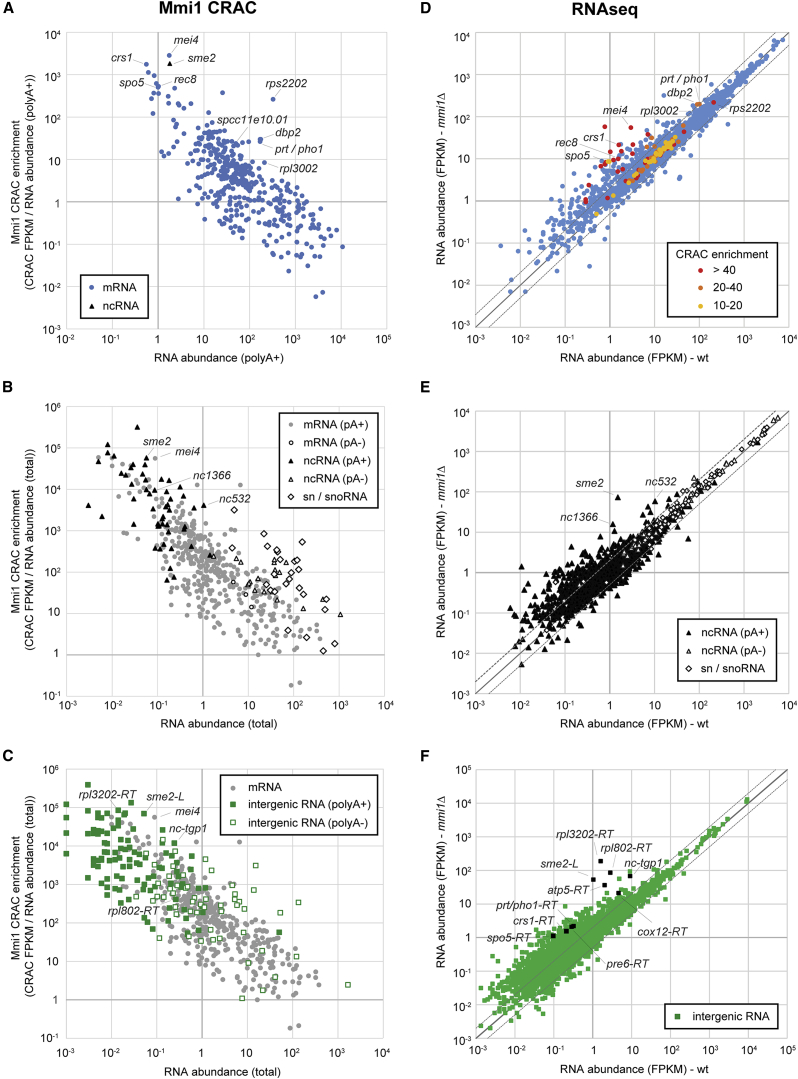
The Role of Mmi1 in Regulation of the Transcriptome (A) Mmi1 CRAC enrichment for mRNAs plotted versus RNA abundance (oligo-dT-selected; Marguerat et al., 2012). Several known (*mei4*, *crs1*, *spo5*, and *rec8*) and selected additional Mmi1 mRNA targets are indicated. The black triangle denotes *sme2* ncRNA. (B) Mmi1 CRAC enrichment plotted versus total RNA abundance (Marguerat et al., 2012). Circles denote mRNAs, triangles ncRNAs, and diamonds sn/snoRNAs. Open and filled markers denote pA− and pA+ transcripts, respectively. Several known (*mei4* and *sme2*) and selected additional Mmi1 ncRNA targets are indicated. (C) Mmi1 CRAC enrichment plotted versus total RNA abundance (Marguerat et al., 2012). Circles denote mRNAs and squares intergenic sequences without annotated features. Open and filled markers denote pA− and pA+ transcripts, respectively. Intergenic regions containing the non-annotated ncRNAs *sme2-L* and *nc-tgp1* or 3′-extended reads (RT) downstream of the indicated genes are labeled. (D–F) RNA-seq analysis of all mRNAs (D), ncRNAs (E), or intergenic regions without annotated features (F) in the WT and *mmi1Δ*. The expression of genes that lie outside of the area indicated by the dashed lines changes >2-fold. Individual transcripts are indicated. See also Figures S2 and S3.

To evaluate the significance of the observed binding events, we normalized all uniquely mapped cDNA counts to the abundance of the target mRNA using polyA+ sequencing data (Marguerat et al., 2012; Table S2). Befittingly, low-abundance transcripts were particularly enriched in Mmi1 CRAC (Figure 2A). For example, the highly unstable meiotic mRNAs *mei4*, *crs1*, *spo5*, and *rec8* were among the most enriched mRNAs. *sme2*, a well-characterized ncRNA target of Mmi1 (Chen et al., 2011, St-André et al., 2010, Yamashita et al., 2012), clustered together with Mmi1-regulated meiotic mRNAs (Figure 2A).

Because not all ncRNAs are polyadenylated, we recalculated CRAC enrichment using published abundance data for total RNA (Marguerat et al., 2012). Strikingly, ncRNAs fall into two distinct clusters that correlated with the presence or absence of pA tails (Figure 2B). The classification into pA-containing (pA+) or non-polyadenylated (pA−) was based on the relative enrichment of the RNA in the oligo-dT-selected fraction versus the total RNA transcriptome (cutoff, 5.0-fold; Marguerat et al., 2012). The results showed that highly enriched ncRNAs in the Mmi1 CRAC data were generally low-abundance and pA+. Conversely, high-abundance, pA− ncRNAs were generally underrepresented in the CRAC data. Intergenic transcripts could also be divided into pA+ and pA− populations (Figure 2C). Intergenic transcripts were most highly enriched in the CRAC experiment, followed by ncRNAs and mRNAs, whereas rRNA enrichment in CRAC was very low (Figure S2A). tRNAs were not included in this analysis because not enough reads could be mapped uniquely.

### The Role of Mmi1 in Transcriptome Regulation

Analysis of our RNA-seq data revealed a large number of differentially expressed genes (Figures 2D–2F). 445 transcripts were downregulated significantly in *mmi1Δ* (>1.5-fold, p < 0.05; Table S3). Of these, 159 were ncRNAs (36%), 147 mRNAs (33%), 135 derived from intergenic regions (30%), and the remaining 1% sn/snoRNAs or pseudogenes. 1,610 transcripts were upregulated significantly in *mmi1Δ* (>1.5-fold, p < 0.05; Table S3), 1,153 of which derived from intergenic regions (72%), 221 were mRNAs (14%), and 210 ncRNAs (13%). The remaining were sn/snoRNAs, tRNAs, or pseudogenes. Transcripts highly enriched in the CRAC data were upregulated frequently in *mmi1Δ* (Figure 2D and S2B). To verify some of our findings, we performed northern blot (NB) or RT-PCR on several transcripts bound by Mmi1, including *rpl3002*, which encodes the ribosomal protein L30, and several ncRNAs (*nc532*, *nc1366*, *nc-tgp1* [not annotated, part of the intergenic feature INT_0_2957], and *nc-spcc11e10.01* [annotated as the extended 5′ UTR of *spcc11e10.01*]) (Figures S2C–S2E). Strikingly, Mmi1 frequently crosslinked with transcripts derived from intergenic regions, which also tended to be upregulated in *mmi1Δ*. This included some non-annotated ncRNAs like *nc-tgp1* or *sme2-L*, a 1.5-kb 3′-extended isoform of *sme2* (Yamashita et al., 2012; Figure 2F). In the majority of cases, however, the increased intergenic signal most likely represented 3′-extended transcripts. These fell into (at least) the following three different classes. (1) Increased reads downstream of genes that were strongly upregulated in *mmi1Δ*; for example, *crs1*, *spo5*, and *prt/pho1* (Figures 2D and 2F). (2) Increased reads downstream of genes of which the overall transcript levels did not change in *mmi1Δ*; for example, *pre6* (Figure 2F and S3A). Generally, these 3′-extended transcripts were at very low levels compared with processed transcripts, suggesting that Mmi1 might be involved in the degradation of read-through products resulting from leaky transcription termination. (3) A small number of genes where we could detect a decrease in reads over the gene body but increased transcription downstream of the 3′ end of the gene, indicative of a transcription termination defect. These included *cox12*, *rpl3202*, *rpl802*, *rps1102*, and *rps2202* (Figure 2F and S3B; Table S3). What predisposes this small subset of genes to defective termination in *mmi1Δ* remains to be determined. In addition, we detected 3′-extended snoR69b in *mmi1Δ* (Figure S3C). cDNA reads from this snoRNA were also present in the Mmi1 CRAC data, indicating that Mmi1 might be involved in its 3′ processing. However, we did not find evidence for a general role for Mmi1 in 3′ processing of sn/snoRNAs.

Interestingly, for ∼100 intron-containing genes, we observed an increase in 5′ ss exon-intron boundary reads in *mmi1Δ* (>2-fold, per million reads) (Table S3). These results can be explained by stabilization of the pre-mRNA, a splicing defect, or a combination of both. To distinguish between these possibilities, we analyzed intron/exon fragments per kilobase of transcript per million reads (FPKM) ratios for intron-containing genes in the RNA-seq data (Figure S3D), which provided an indication of changes in splicing efficiency in the *mmi1Δ* strain. In the majority of cases, the intron/exon FPKM ratio did not change noticeably, suggesting that the increase in 5′ ss exon-intron boundary reads was probably the result of increased stabilization of the pre-mRNA (Figure S3D). However, for a small number of genes, we could detect an increase both in 5′ ss exon boundary reads and in the intron/exon FPKM ratio in the *mmi1Δ* strain, indicative of a splicing defect (Figure S3D). This included the Mmi1 target *rps2202*. However, it should be noted that the splicing defect in *rps2202* appears to be independent of recruitment of Mmi1 to the transcript because it was not observed upon mutation of the Mmi1-binding site (see below). Why specifically these transcripts show splicing defects in *mmi1Δ* remains unclear. Our data strongly suggest that the main function of Mmi1 is RNA degradation. Only a minor portion of the transcriptome depends on Mmi1 for RNA processing (splicing and 3′ end formation), and it is not clear whether these effects are direct.

Finally, 57% of mRNAs upregulated in an MTREC mutant (*red1Δ*) (Sugiyama and Sugioka-Sugiyama, 2011) were also upregulated in *mmi1Δ* (fold change >1.5, p < 0.05; Table S3). The MTREC-associated protein Pab2 has also been implicated in the downregulation of meiotic genes via Mmi1 (St-André et al., 2010). In addition, Pab2 has been found to act in the selective degradation of pre-mRNAs (Lemieux et al., 2011). Strikingly, roughly half of all Pab2-regulated intron-containing mRNAs were also upregulated significantly in *mmi1Δ* (Table S3), which suggests that Mmi1 binding could be an important determinant for Pab2-mediated pre-mRNA turnover.

### Mmi1 Binding to Introns Regulates the Accumulation of Spliced Product

**Figure 3:**
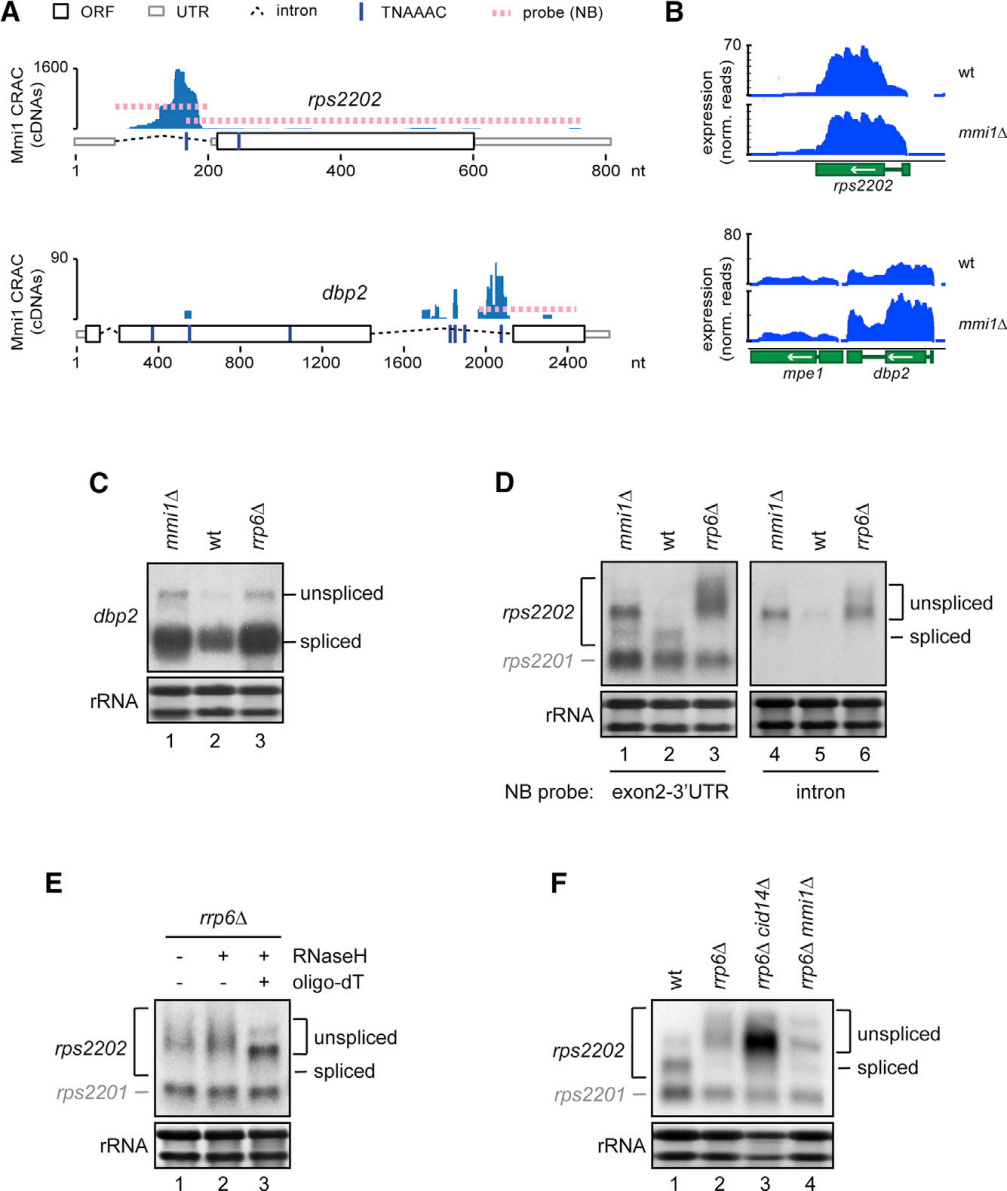
Mmi1 Binding to Introns Regulates the Accumulation of Spliced Product (A) Mmi1 CRAC cDNA reads over the intron-containing genes *dbp2* and *rps2202*. The positions of the TNAAAC motifs and position of probes used for NB in (C) and (D) are indicated. (B) RNA-seq analysis of the Mmi1 target genes shown in (A). (C) *dbp2* northern blot analysis. A DNA probe against exon 3 was used (see A). (D) *rps2202* northern analysis. Bands corresponding to spliced and unspliced *rps2202* and the paralogue *rps2201* are indicated. Because of high sequence conservation, the intron-less paralogue *rps2201* is also detected (Figure S4C). Left: a DNA probe against exon 2 was used. Right: a strand-specific RNA probe against the intron was used (see A). (E) *rps2202* northern blot analysis on total RNA treated or not treated with RNaseH in the presence or absence of oligo-dT to cleave pA tails. (F) *rps2202* northern blot analysis. See also Figure S4.

Intriguingly, among the transcripts with the highest number of cDNA reads in the Mmi1 CRAC data were *dbp2* and *rps2202*, where Mmi1 is bound within intronic regions (Figure 3A; Table S4). This suggested that Mmi1 could specifically regulate levels of the unspliced pre-mRNA, whereas the mRNA is not expected to be targeted because the Mmi1 binding site is removed by splicing. Indeed, we observed an increase in intronic reads in *mmi1Δ* (Figure 3B) and could show accumulation of *rps2202* and *dbp2* pre-mRNA in *mmi1Δ* and *rrp6Δ* by NB (Figures 3C and 3D) or RT-PCR (Figure S4A). Pre-mRNA accumulation was also observed when *pab2* or *red1* was deleted (Figure S4B, lanes 6 and 7), which are known to act in the turnover of meiotic mRNAs (Egan et al., 2014, Lee et al., 2013, St-André et al., 2010, Sugiyama and Sugioka-Sugiyama, 2011). Unexpectedly, levels of the spliced *dbp2* transcript were also increased strongly in *mmi1Δ*, suggesting that Mmi1 binding regulates mRNA levels, although Mmi1 cross-linking to regions outside of the intron was negligible (Figures 3A and 3C). In contrast, *rps2202* splicing was inhibited in *rrp6Δ* and, to some extent, in *mmi1Δ* (Figure 3D and S4A). Intron hyperretention (hyper-IR) resulted in decreased levels of mRNA and was observed in other nuclear surveillance mutants (*dis3-54* and *cid14Δ*) but not in *pab2Δ* or *red1Δ* (Figure S4B, lanes 4–7). The *rrp6Δ* strain also accumulated 3′-extended *rps2202* species that were detected as a smear above the major pre-mRNA band (Figure 3D). This can be indicative of hyperadenylation, which has been reported to occur on several meiotic Mmi1 targets in exosome mutants (Chen et al., 2011, Yamanaka et al., 2010, Zhou et al., 2015). Consistent with this idea, the smear disappeared upon RNase H digestion in the presence of oligo-dT (Figure 3E). Hyperadenylation of meiotic mRNAs depends on Mmi1 but does not require the TRAMP component *cid14* (Yamanaka et al., 2010), and we found the same for *rps2202* (Figure 3F). However, *dbp2* and *rps2202* pre-mRNAs were stabilized in *cid14Δ* (Figure S4B, lane 5). Also, the double mutant *cid14Δ rrp6Δ* accumulated more *rps2202* pre-mRNA than the single mutants (the strain is very sick; Figure 3F, lanes 2 and 3). This suggests that *cid14* is involved in pre-mRNA quality control in *S. pombe*.

### *dbp2* Intron 2 Negatively Regulates Levels of the Spliced mRNA

**Figure 4:**
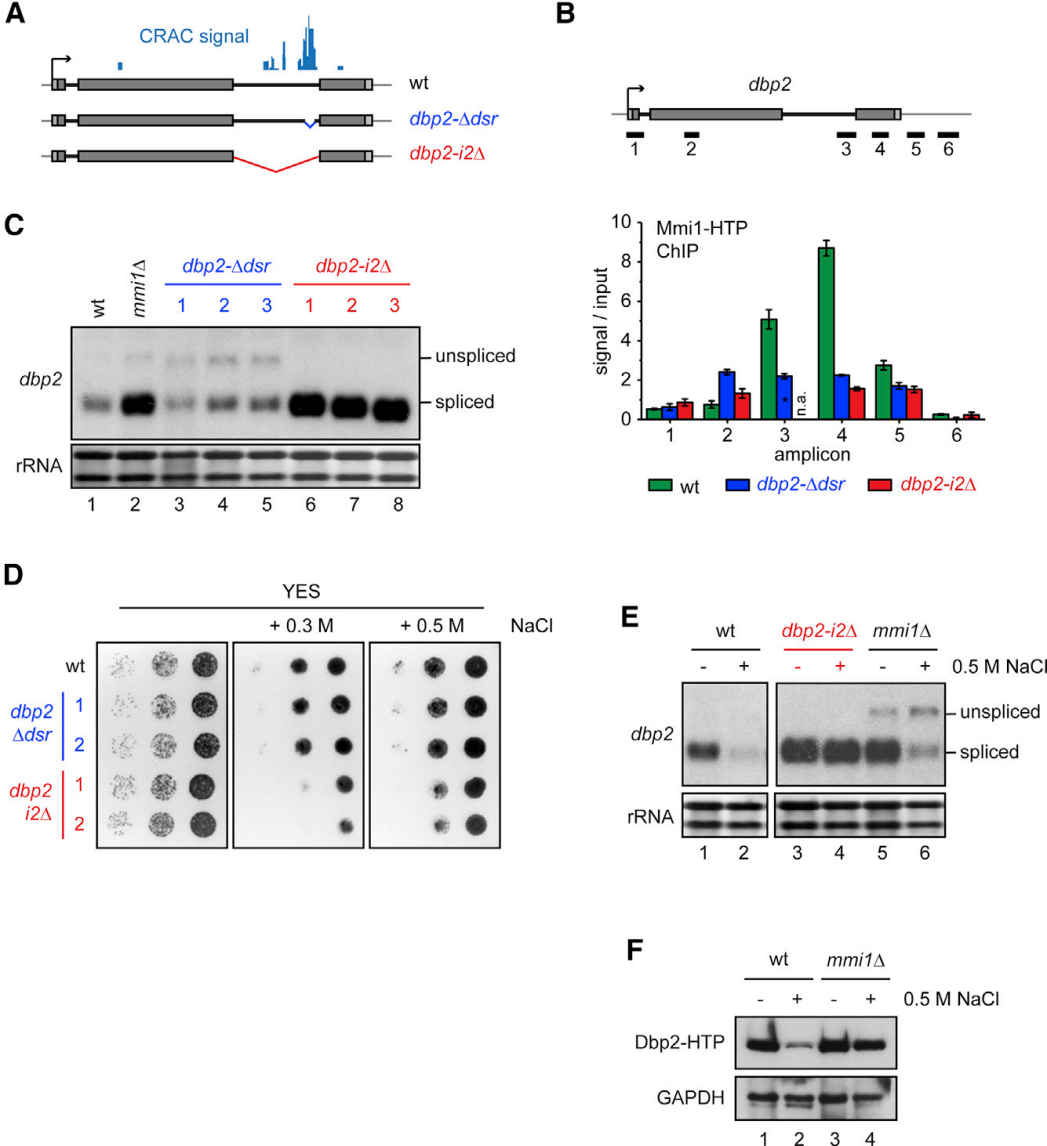
*dbp2* Intron 2 Is Involved in Negative Expression Regulation (A) Schematic of the constructs used in (B)–(E). The position of the Mmi1 CRAC signal in the WT is indicated. (B) ChIP analysis of Mmi1-HTP recruitment across the *dbp2* locus. The positions of the amplicons are indicated above the bar plot. Error bars indicate SEM of at least two biological replicates. Note that amplicon 3 is shorter in *dbp2-Δdsr* and, therefore, cannot be compared with the results for the other strains. (C) *dbp2* northern blot analysis. For *dbp2-Δdsr* and *dbp2-i2Δ*, we included three separate clones. (D) Serial dilution of the indicated yeast strains on YES plates with various salt concentrations, incubated at 30°C. Note that the plates were photographed after different incubation times (1–3 days), depending on cell growth. (E) *dbp2* northern blot analysis of RNA extracted from cells grown for 24 hr in YES with or without added NaCl. Note that the two panels are taken from different blots. For direct comparison of the WT and *mmi1Δ*, refer to Figure S5F. (F) Western blot analysis of whole-cell extracts from strains expressing Dbp2-HTP and grown for 24 hr in YES with or without added NaCl. See also Figure S5.

Next, we wanted to investigate how Mmi1 regulates *dbp2* expression. *dbp2* encodes a conserved DEAD box helicase with functions in RNA metabolism and harbors two introns. Interestingly, intron 2, which contains the Mmi1-binding sites, is conserved in *S. cerevisiae* and higher eukaryotes with respect to its position and length. Splicing efficiency has been reported to be low for this intron in various organisms, including *S. pombe*, where it induces Pab2-dependent decay of the pre-mRNA when transplanted into a different gene (Barta and Iggo, 1995, Iggo et al., 1991, Lemieux et al., 2011, Moore et al., 2011). In agreement with this, pre-mRNA with a retained intron 2 was observed in the wild-type (WT) (Figure S5A). This is unlike intron 1, which is spliced efficiently (Lemieux et al., 2011; Figure S5A). To test whether intron 2 is involved in the regulation of *dbp2* levels, we generated an intron 2 deletion mutant (*dbp2-i2Δ*; Figure 4A). Although this mutant failed to recruit Mmi1 to *dbp2*, binding of Mmi1 to *rps2202* was unaffected (Figure 4B and S5B), demonstrating that Mmi1 preferentially binds the *dbp2* intron. Notably, levels of *dbp2*-*i2Δ* mRNA were increased strongly compared with the WT transcript (Figure 4C, lanes 6–8), reminiscent of what we observed in the *mmi1Δ* strain (Figure 4C, lane 2).

Because deletion of the entire intron can remove additional regulatory elements that are independent of Mmi1, we deleted a 73-base pair (bp) region within the intron for which we had detected the strongest cross-linking to Mmi1 (*dbp2-Δdsr*; Figure 4A). Although Mmi1 recruitment to *dbp2* was reduced strongly and the pre-mRNA stabilized, this mutation had little influence on spliced RNA levels, suggesting that additional destabilization elements are present in the intron, perhaps other Mmi1 binding sites not detected in CRAC (Figures 4B and 4C, lanes 3–5).

Given that a futile cycle of synthesis and decay is energy-consuming, we reasoned that the increased energy expenditure associated with the *dbp2* intron may be evolutionarily tolerated because the presence of the intron (or specific features within the intron, such as the Mmi1-binding motifs) could confer an advantage to the cell; for example, by facilitating regulation of gene expression. This prompted us to investigate whether removal of the intron would be detrimental to growth under certain conditions. To test this, we serially diluted cells expressing WT *dbp2* or *dbp2-i2Δ* onto different growth media. In *S. cerevisiae*, Dbp2 responds to glucose depletion (Beck et al., 2014). However, we did not observe any differences in growth on plates with different carbon sources (Figure S5C). In contrast, cells expressing *dbp2-i2Δ* grew slower in medium containing elevated salt concentrations (Figure 4D). Under these conditions, levels of WT *dbp2* were dramatically reduced (Figure 4E, lane 2), whereas *dbp2-i2Δ* levels remained constitutively high (Figure 4E, lane 4). Pol II occupancy at the *dbp2* gene locus was decreased under high-salt conditions (Figure S5D). Importantly, however, deletion of intron 2 had no effect on Pol II levels compared with the WT strain, in stark contrast to the pronounced differences in RNA levels. This suggested that the intron exerts its influence through a post-transcriptional mechanism. Intron 2 was also required for *dbp2* destabilization under other stress conditions, including DNA damage (Figure S5E, lanes 2 and 4). Therefore, we hypothesized that increased retention of intron 2 and targeted removal of the unspliced RNA by the Mmi1/exosome pathway negatively regulates *dbp2* levels in response to stress. In agreement with increased IR, in *mmi1Δ*, more pre-mRNA was stabilized under high-salt conditions, and less fully spliced *dbp2* was generated (Figure 4E, lane 6). Also, negative regulation in response to salt was impaired in *mmi1Δ* (Figure S5F), which was best observed at the protein level (Figure 4F). This strongly supported the notion that Mmi1 recruitment to introns can amplify the effects of inefficient splicing and suggests that fission yeast employs IR coupled to rapid nuclear RNA decay to tightly control Dbp2 expression.

### Recruitment of Mmi1 to the *rps2202* Intron Occurs in the Context of Paralogue-Dependent Negative Splicing Regulation

**Figure 5:**
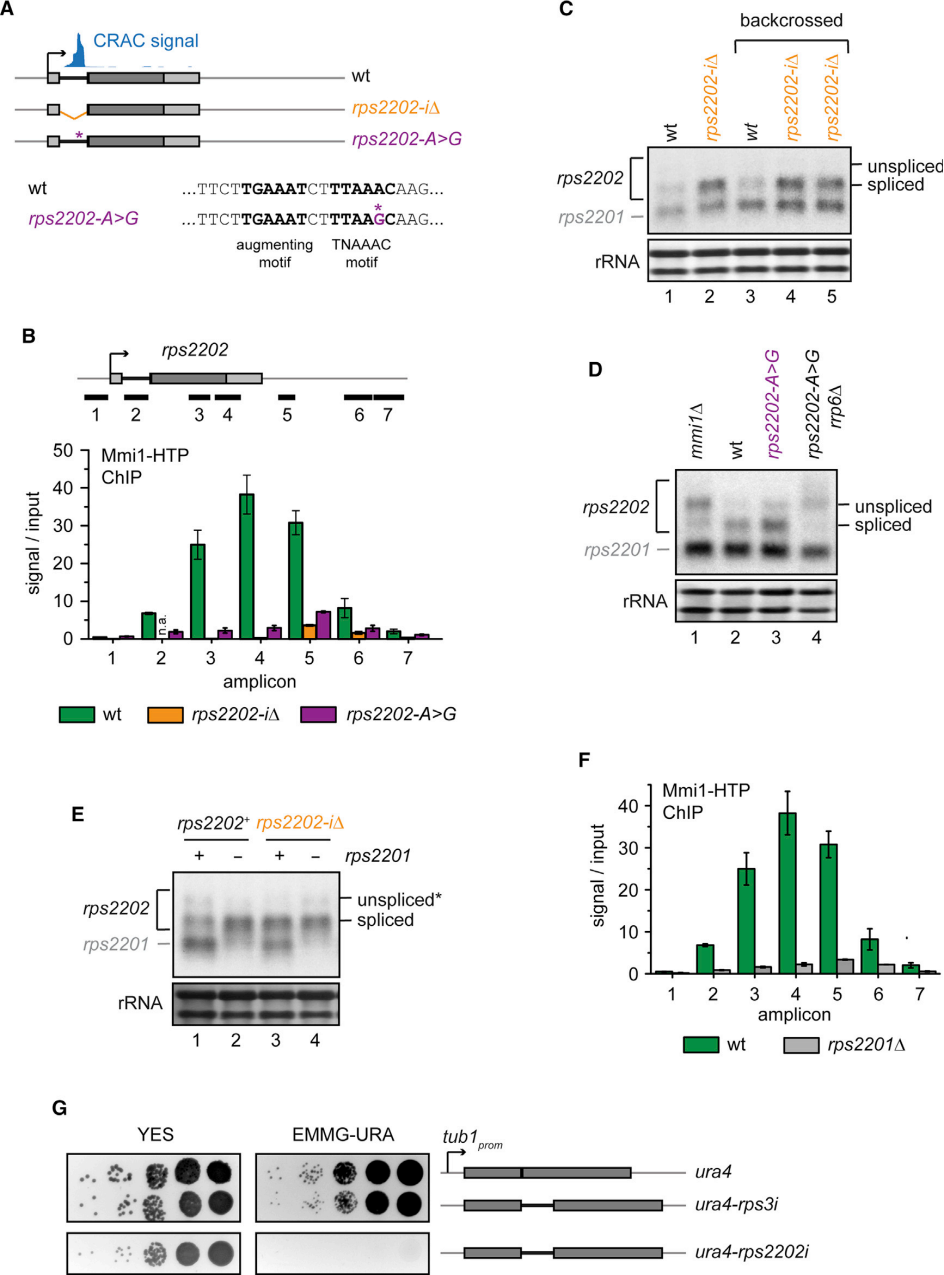
Recruitment of Mmi1 to the *rps2202* Intron Occurs in a Context of Paralogue-Dependent Negative Splicing Regulation (A) Schematic of the constructs used in (B)–(E). *rps2202-A>G* harbors a single base pair mutation in the TNAAAC motif. The position of the Mmi1 CRAC signal in the WT is indicated. (B) ChIP analysis of Mmi1-HTP recruitment across *rps2202*. The positions of the amplicons are indicated above the bar plot. Error bars indicate SEM of at least two biological replicates. (C–E) *rps2202* northern blot analysis. Because of high sequence conservation, the paralogue *rps2201* is also detected. (C) The *rps2202*-*iΔ* mutant was backcrossed into the WT background to verify that the phenotype co-segregated with the mutation (lanes 3–5). (D) *rps2202-A>G* was crossed with *rrp6Δ* to generate the double mutant (lane 4). (E) Note that the unspliced precursor co-migrates with an extended spliced form (^∗^) that is also present in *rps2202*-*iΔ*. No intron-containing band is detected in *rps2202*-*iΔ* by RT-PCR (Figure S6C). (F) ChIP analysis of Mmi1-HTP recruitment across *rps2202*. Error bars indicate SEM of at least two biological replicates. (G) Serial dilution of yeast strains with an *ura4* reporter (intron-free or containing introns of *rps3* or *rps2202*) driven by the *tub1* promoter integrated into the *leu1* locus grown on plates lacking uracil (EMMG-URA). See also Figure S6.

As shown above, Mmi1 also strongly binds the intron of *rps2202*, which encodes the highly conserved ribosomal protein (RP) S15a (Figure 3A). Unspliced *rps2202* was detectable in the parental strain, suggesting that splicing is inefficient under normal growth conditions (Figure S4A). To test whether this intron, too, contributes to the regulation of mRNA levels, we constructed a mutant in which the intron was deleted (*rps2202*-*iΔ*; Figure 5A). The deletion strongly affected Mmi1 recruitment to *rps2202* but not to a control gene (*dbp2*) (Figures 5B and S6A) and resulted in increased levels of *rps2202* (Figures 5C and S6C). This suggested that the presence of the intron negatively regulates *rps2202* expression.

In *mmi1Δ* and various nuclear surveillance mutants, we observed hyper-IR of this intron (Figures 3D and S4B). This could indicate that Mmi1 binding is required for splicing. However, a single base pair mutation in the tandem Mmi1-binding site did not induce hyper-IR, although co-transcriptional Mmi1 recruitment was reduced strongly (*rps2202-A>G*; Figures 5B and S6B). Rather, as for *rps2202*-*iΔ*, *rps2202* levels were increased in this strain (Figure 5D, lane 3). We take this as an indication that hyper-IR may not be directly linked to Mmi1 recruitment to the locus but could be caused by the misregulation of an unknown factor in nuclear surveillance mutants. In addition, *rps2202-A>G* did not rescue hyper-IR in *rrp6Δ* nor, interestingly, hyperadenylation of the pre-mRNA (Figure 5D, lane 4), suggesting that hyperadenylation may not be strictly dependent on recruitment of Mmi1 to the gene.

Based on the phenotypes of *rps2202*-*iΔ* and *rps2202-A>G*, we conclude that, similar to *dbp2*, the poorly spliced intron limits *rps2202* expression through Mmi1/exosome-dependent decay. For several other RP genes it has been reported that their levels are regulated by paralogue-dependent inhibition of splicing (Lemieux et al., 2011, Macías et al., 2008, Plocik and Guthrie, 2012, Vilardell and Warner, 1997). The *S. pombe* genome harbors a second copy of S15a, *rps2201*, which encodes the identical protein but contains no intron. With our NB probes we detected both *rps2201* and *rps2202* because of the high sequence conservation (Figures 5C and 5D). This raised the possibility that S15a levels are controlled by a similar regulatory feedback mechanism. Accordingly, deletion of *rps2201* resulted in increased *rps2202* expression, comparable with *rps2202*-*iΔ* (Figure 5E, lanes 2 and 3). In both mutants, Pol II levels over *rps2202* were unchanged, making regulation at the transcriptional level unlikely (Figure S6D). Also, deletion of *rps2201* in the intron-less strain did not lead to a further increase in *rps2202* levels, compatible with the idea that Rps2201 affects *rps2202* levels by modulating its splicing (Figure 5E, lane 4). To examine whether Rps2201 regulates IR in *rps2202*, we compared splicing efficiencies in the Pol II-bound RNA fraction of *rps2201Δ* and the WT to accurately evaluate levels of the unstable precursor. Almost no spliced *rps2202* was detected in *rrp6Δ*, confirming the occurrence of hyper-IR in this strain (Figure S6E, lane 5). Importantly, upon deletion of *rps2201*, higher levels of the spliced form were associated with Pol II (Figure S6E, lane 6), indicative of increased rates of splicing. Interestingly, deletion of *rps2201* also rescued *rps2202* hyper-IR in nuclear surveillance mutants (Figure S6F, lanes 3, 5, 7, and 9). We conclude that IR in *rps2202* depends on S15a levels and propose that negative regulation of *rps2202* splicing helps to tightly control S15a expression through the Mmi1/exosome pathway. Importantly, this regulation provided us with a tool to assess the influence of splicing rates on the recruitment of Mmi1 to intronic sites. Strikingly, Mmi1 recruitment to *rps2202* was reduced dramatically in the absence of Rps2201, suggesting a kinetic competition between splicing and Mmi1 recruitment (Figure 5F).

To examine whether the presence of the *rps2202* intron was sufficient to destabilize RNA, we inserted the intron into an *ura4* reporter driven by the *tub1* promoter, which enables growth on selective media lacking uracil (Edinburgh minimal medium with glutamate without uracil [EMMG-URA]). As negative controls, we either integrated intron-less *ura4* or a construct that contained the *rps3* intron, which is spliced efficiently (Lemieux et al., 2011). The presence of the *rps3* intron within *ura4* had a very mild effect on growth on EMMG-URA, suggesting that the *rps3* intron is indeed spliced efficiently. However, cells that expressed the *ura4-rps2202i* reporter were unable to grow (Figure 5G). Similarly, hardly any spliced mRNA could be detected when the *rps2202* intron was inserted into another reporter, EGFP (Figures S6G, lane 2, and S6H). However, splicing was enabled when the paralogue *rps2201* was deleted (Figure S6G, lane 4), again confirming that the *rps2202* intron is subject to S15a level-dependent negative splicing regulation. We conclude that the *rps2202* intron can repress expression when integrated into a reporter construct and retains its ability to be regulated.

## Discussion

Orchestrated changes in RNA stability play an important role in transitions between different metabolic states, and widespread intron retention has been proposed to be an important trigger for RNA decay in response to stress (Bergkessel et al., 2011, Gagnon et al., 2015, Marguerat et al., 2014, Pleiss et al., 2007). The importance of this pathway has been underscored by a systematic study that analyzed the effect of intron deletions on cell fitness in *S. cerevisiae*, which frequently resulted in reduced survival under stress (Parenteau et al., 2011). However, a substantial fraction of pre-mRNAs is degraded by the nuclear exosome even under optimal growth conditions, resulting in constant removal of newly transcribed material (Gudipati et al., 2012, Schneider et al., 2012). Why cells employ such a seemingly energy-expensive system has been a matter of debate. It has been suggested that quality control could be favored kinetically to minimize the risk of toxicity associated with defects in processing. But why should RNA processing be that error-prone in the first place?

**Figure 6:**
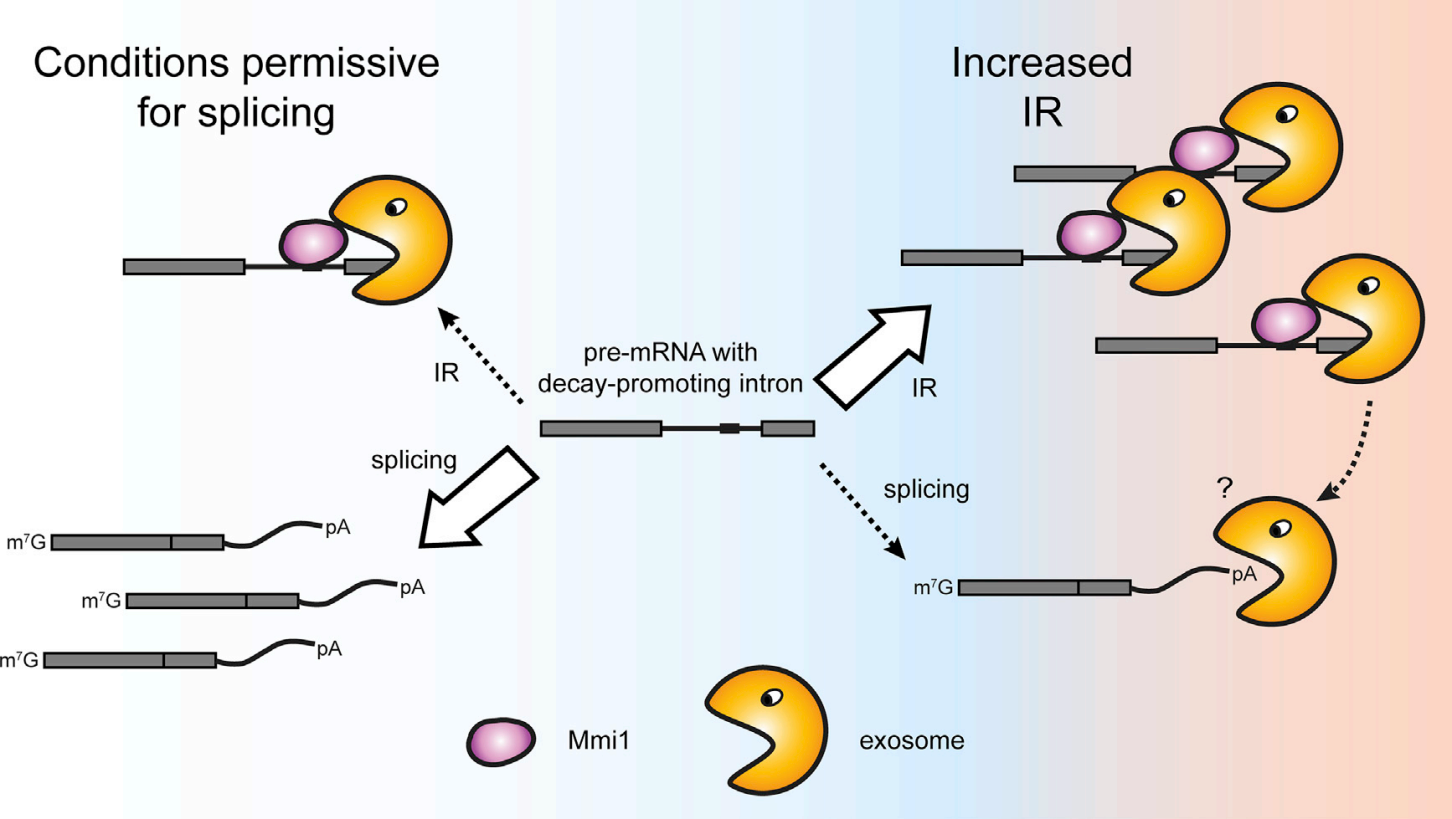
Model of Regulation of Gene Expression by Decay-Promoting Introns that Activate Nuclear Surveillance Decay-promoting introns contained in fission yeast pre-mRNAs harbor Mmi1-binding sites that have the ability to induce fast transcript turnover. Although fast splicing prevents the recruitment of Mmi1 and the activation of nuclear surveillance, increased IR under stress conditions or in the presence of a specific splicing inhibitor results in reduced gene expression. It is currently unclear whether intron-dependent recruitment of the exosome to the gene locus could also trigger degradation of fully spliced molecules that are retained in the same nuclear compartment.

Both *dbp2* and *rps2202* transcripts are spliced inefficiently under standard laboratory conditions, and the pre-mRNAs are turned over rapidly, resulting in a futile cycle of RNA synthesis and decay. Importantly, however, the rates with which these introns are spliced are not constant but change according to the conditions. Fast intron removal precludes Mmi1 recruitment and results in increased gene expression. It seems likely that fission yeast maintains these introns, although this is energy-consuming, because they facilitate the regulation of gene expression and ensure cell survival at times of changing conditions (Figure 6). Importantly, we find that the selective degradation of these pre-mRNAs involves active targeting of the RNA decay machinery to the intron-containing transcript. This contrasts with the current view in the field, which has been focused on the identification of a default pathway that could, for example, respond to stalled spliceosomes (Lee et al., 2013, Nag and Steitz, 2012, Zhou et al., 2015). To differentiate between both mechanisms, we propose to introduce the concept of decay-promoting introns that actively induce RNA degradation when retained. This category could also include introns that contain RNase III cleavage sites that elicit RNase III-mediated decay (RMD) (Danin-Kreiselman et al., 2003, Roy and Chanfreau, 2014). Nuclear RNA turnover induced by decay-promoting introns complements other post-transcriptional gene regulatory pathways, such as alternative splicing coupled with NMD or spliceosome-mediated decay (SMD), where non-productive splicing generates unstable products that are turned over rapidly by the exosome (Volanakis et al., 2013).

Currently, we do not know which factors negatively regulate splicing of *dbp2*, and we are only aware of conditions that decrease the low splicing efficiency even further (high salt). Intriguingly, the pathway may be conserved. DDX5/p68, for example, the human homolog of *dbp2*, also contains the conserved intron (Moore et al., 2011). In *S. cerevisiae*, IR of the *dbp2* intron is relieved in a *dbp2* point mutant (helicase-dead), suggesting that the gene may be autoregulated (Barta and Iggo, 1995). In *S. pombe*, we could detect Dbp2 across the *dbp2* locus (data not shown). Therefore, autoregulation of *dbp2* splicing in *S. pombe* clearly remains a possibility. If true, then this would suggest that IR could be alleviated under conditions where the pool of free Dbp2 is decreased or its access to the nucleus is restricted, as occurs in *S. cerevisiae* upon glucose depletion (Beck et al., 2014).

For *rps2202*, we find that splicing is influenced negatively by its paralogue Rps2201, suggestive of tight control of protein homeostasis. There is precedence for paralogue-dependent regulation of splicing: *S. cerevisiae* Rpl30p is known to inhibit splicing of its own mRNA by impeding the progression of splicing (Macías et al., 2008, Vilardell et al., 2000). Fission yeast possesses two copies of *RPL30*, and here the protein product of one paralogue (*rpl30-1*) negatively influences splicing of the other (*rpl30-2*) (Lemieux et al., 2011). Interestingly, *rpl30-2* is also among the Mmi1 targets identified by our CRAC and is upregulated in *mmi1Δ* (Figure 2D). More reports of auto- or cross-paralogue inhibition of splicing in different species exist, and this mode of regulation appears to be particularly widespread among ribosomal protein genes (Fewell and Woolford, 1999, Lemieux et al., 2011, Malygin et al., 2007, Parenteau et al., 2011, Plocik and Guthrie, 2012). Interestingly, RP genes are enriched significantly in our Mmi1 CRAC (gene ontology [GO] term enrichment for “cytoplasmic translation” [GO:0002181] 47/238 [p = 2.52 × 10^−8^] and for “translation” [GO:0006412] 54/350 [p = 5.32 × 10^−6^]). Therefore, Mmi1 could have a broader role in the regulation of ribosome biogenesis.

Several points remain to be addressed in the future. What causes hyper-IR in *rps2202* in nuclear surveillance mutants? Globally, only a handful of genes display hyper-IR in *mmi1Δ* (Figure S3D), and it will be an interesting direction for future research to find out whether they are regulated by common factors. Multiple mRNAs identified in Mmi1 CRAC and/or deregulated in *mmi1Δ* encode proteins that have been linked to RNA biogenesis. One example is Mlo3 (a homolog of the *S. cerevisiae* export factor Yra1), which associates with TRAMP to suppress RNA transcribed from heterochromatic loci (Bühler et al., 2007, Zhang et al., 2011). In *mmi1Δ*, *mlo3* transcription termination is defective (Table S3; data not shown), and its reduced expression could be a possible cause of the pleiotropic effects observed in *mmi1* mutants.

Based on our Mmi1 CRAC experiment, we identified only two dozen introns that could qualify as decay-promoting (Table S4). However, our data clearly suggest that fast splicing precludes Mmi1 recruitment (Figure 5F). Therefore, other introns may splice too rapidly under normal growth conditions to be bound by Mmi1 but may have the potential to do so should the rate of splicing decrease. In accordance with this idea, 4.8% of *S. pombe* introns contain at least one TNAAAC motif. Contrary to what was observed in reporter assays, where at least six repeats of the motif were needed for efficient silencing (Yamashita et al., 2012), our data suggest that a single intronic TNAAAC motif is sufficient to commit *rps2202* to decay. On the other hand, we did not observe Mmi1 binding to every possible motif. These observations point to additional requirements for Mmi1 binding, such as a specific RNA fold or the presence of accessory proteins. Alternatively, as has been shown recently for other YTH domain proteins, Mmi1 affinity may be modulated by N6-methylated adenosines, an RNA modification that has been linked to alternative splicing (Dominissini et al., 2012, Wang et al., 2014). It will be an interesting future direction of research to study whether Mmi1 binding could be regulated by this RNA modification.

## Experimental Procedures

Additional detailed protocols are available in the Supplemental Experimental Procedures. All oligonucleotides used in this study are listed in Table S5.

### Yeast Strains and Manipulations

All *S. pombe* strains used in this study are listed in Table S6. Standard methods were used for cell growth and genetic manipulations (Moreno et al., 1991). Cells were grown in yeast extract with supplements (YES) at 30°C unless indicated otherwise.

### RNA Sequencing

Libraries were sequenced on the Illumina HiSeq 2500 platform. Library preparation and analysis are described in the Supplemental Experimental Procedures.

### Preparation of RNA and RT-PCR

RNA extractions were performed as described previously (Vasiljeva and Buratowski, 2006). Reverse transcription was carried out on DNase-treated total RNA using gene-specific primers followed by PCR. RT controls were performed for each experiment (data not shown).

### Northern Blot

NB experiments were performed as described previously (Vasiljeva and Buratowski, 2006). RNA was resolved on 1.2% agarose gels. For strand-specific NB, digoxigenin (DIG)-labeled probes were in vitro-transcribed with MAXIscript (Ambion) and detected using the DIG system (Roche). RNase H treatment in the presence of oligo-dT was carried out on 10 μg total RNA as described previously (Yamanaka et al., 2010). In all cases, methylene blue staining of ribosomal bands served as a loading control.

### Chromatin Immunoprecipitation

Chromatin immunoprecipitation (ChIP) assays were performed as described previously (Shah et al., 2014) and quantified with qPCR using SensiMix SYBR (Bioline). Immunoprecipitations (IPs) were conducted with either rabbit IgG agarose (Sigma) or antibody against Rpb1 (Millipore, 8WG16) coupled to protein G Dynabeads (Life Technologies). The values shown correspond to the ChIP signal above the non-tagged background (for immunoglobulin G [IgG]) over the input relative to a control gene (*fbp1*). Error bars represent SEM of at least two biological replicates.

## Author Contributions

C.K., S.G., and L.V. designed experiments; C.K., S.W., M.P., S.S., and S.G. performed experiments; C.K. and S.G. analyzed data; C.K., S.G., and L.V. wrote the manuscript. All authors edited the manuscript.

## Accession Numbers

The accession number for the raw (fastq) and processed sequencing data reported in this paper is GEO: GSE73144.

## Supplemental Information

Document S1. Supplemental Experimental Procedures, Figures S1–S6, and Tables S1 and S4

Document S2. Tables S2, S5, and S6 Analysis of Mmi1 CRAC data. Contains data used to generate Figure S1A.

Table S3. Analyses of *mmi1Δ* and WT RNA-Seq Data, Related to Figure 2 Contains analyses of the RNA-seq data used to generate Figure 2, including : FPKMs of RNA-seq data, DESeq2 results of RNA-seq data, protein-coding genes with substantial increase of 3’ extended reads and/or decreased reads over the gene body in mmi1Δ, indicative of transcription termination defect, ratio of exon-intron boundary reads, fold change mmi1Δ /WT RNA-seq for genes upregulated in red1Δ > 2-fold (Sugiyama and Sugioka-Sugiyama, 2011), and fold change mmi1Δ /WT RNA-seq for genes with increased levels of spliced and unspliced mRNA in pab2Δ (Lemieux et al., 2011).

Document S3. Article plus Supplemental Information
