# Supplementary material for: Regulation of mRNA Levels by Decay-Promoting Introns that Recruit the Exosome Specificity Factor Mmi1
Source: Cell Rep. 2015 Dec 6;13(11):2504–15. doi: 10.1016/j.celrep.2015.11.026 (PMC4695336; doi:10.1016/j.celrep.2015.11.026)
Supplement: Document S1. Supplemental Experimental Procedures, Figures S1–S6, and Tables S1 and S4 [file mmc1.pdf]

Cell Reports

Supplemental Information

Regulation of mRNA Levels by Decay-Promoting Introns that Recruit the Exosome Specificity Factor Mmi1

Cornelia Kilchert, Sina Wittmann, Monica Passoni, Sneha Shah, Sander Granneman, and Lidia Vasiljeva

Regulation of mRNA levels by decay-promoting introns that recruit the exosome specificity factor Mmi1

Cornelia Kilchert, Sina Wittmann, Monica Passoni, Sneha Shah, Sander Granneman and Lidia Vasiljeva

SUPPLEMENTAL EXPERIMENTAL PROCEDURES

Strain construction

Genomic mutations were introduced by the pop-in, pop-out method (Gao et al., 2014). DNA fragments containing the desired mutation were generated by 2-step PCR with the oligonucleotides listed in Table S5, inserted into pCR blunt II TOPO (Life Technologies) according to the manufacturer's instructions, and subcloned into pKS-URA4 (Bähler et al., 1998) after BamHI/NotI digestion. Plasmids were linearized with BseRI before transformation into yeast. Positive clones after pop-out on 5-FOA plates were identified by colony PCR and the presence of the mutations was verified by sequencing. Plasmids used as templates for cassette generation were pKS-URA4 (Bähler et al., 1998) for the deletion of *rps2201*, and pBS1479-HTP-KanMX for his_6-TEV-ProtA-tagging, in which the TRP selection marker on pBS1479-HTP (Granneman et al., 2009) was replaced with KanMX. Reporter constructs were generated in a pDUAL derivative (Matsuyama et al., 2004) that contains the *S. pombe tub1* promoter (a kind gift from the Nasmyth lab) and integrated into the *leu1* locus after restriction digest. The *ura4* sequence was taken from pKS-URA4 (Bähler et al., 1998), EGFP from pAD301 (a kind gift from A. Diepold).

CRAC and bioinformatics analyses

S. pombe cells were grown in YES medium to an OD_{600} of 0.5 and UV-irradiated in the Megatron UV cross-linker (Granneman et al., 2011) for 200 seconds. CRAC was performed as previously described (Granneman et al., 2009) with slight modifications: We used App_PE 3' adapter and App_RT oligonucleotide (see Table S5) to make the libraries compatible with paired-end sequencing. The cDNA libraries were PCR amplified using P5 and barcoded P3 oligonucleotides (Table S5) and CRAC libraries were paired-end sequenced on a HiSeq2500 at Edinburgh Genomics, University of Edinburgh. Data from two biological replicates (untagged control and Mmi1-HTP samples) were subsequently merged. Data analyses were performed using the pyCRAC package (Webb et al., 2014) and in-house python scripts. Demultiplexing was performed using pyBarcodeFilter. After trimming adapter sequences from the fastq files, potential PCR duplicates were removed using pyFastqDuplicateRemover and reads with more than 7nt A or T homopolymeric stretches were filtered out. The cDNAs were aligned to the *S. pombe* genomic reference sequence (ASM294v2.19) using Novoalign version 2.05. The cDNAs that mapped to multiple genomic positions were randomly distributed over possible mapping positions (novoalign -r Random flag). Feature counts were generated using pyReadCounters. All analyses were performed on cDNA that mapped to a single genomic position. For the motif analyses, cDNA clusters were generated for reads mapping to protein-coding genes using pyClusterReads (default settings). The resulting GTF output file was subsequently fed to pyMotif to identify enriched 4-8nt k-mers. The sequence logo shown in Figure 1C was prepared using Weblogo (http://weblogo.berkeley.edu/logo.cgi).

RNA-seq and bioinformatics analyses

Total RNA was extracted from three separate cultures of wt and *mmi1Δ* grown to mid-log phase and DNase-treated. Ribodepletion and library preparation were performed by the High-Throughput Genomics Group at the Wellcome Trust Centre for Human Genetics according to the TruSeq protocol. Quality trimming of reads was performed using FlexBar (version 2.5) after which reads were aligned to the *S. pombe* genomic reference sequence (ASM294v2.19) using Novoalign version 2.05. The reads that mapped to multiple genomic positions were randomly distributed over possible mapping positions (novoalign -r Random flag). Feature counts were generated using pyReadCounters from the pyCRAC package (Webb et al., 2014) and differential expression analyses was performed using DESeq2 (Love et al., 2014).

Analysis of splicing efficiencies of Pol II-associated transcripts by RT-PCR

To analyse splicing efficiencies of Pol II-associated transcripts, 1 litre of yeast culture was grown in YES to an OD_{600} of 1.5, harvested and yeast beads shock-frozen in liquid N_2. Beads were ground to a fine powder in the presence of liquid N_2 and the powder thawed slowly at 4°C. An equal volume of TNG buffer (20 mM Tris-HCl, pH8.0, 150 mM NaCl, 10% glycerol, supplemented with 2 mM DTT, fungal protease inhibitor cocktail (Roche), and RNasin (Promega)) and acid-washed glass beads were added, and the mixture vortexed at full speed for 5 x 30 sec pulses. The lysate was transferred to a centrifuge tube with one volume of TNG + 1.5mM $MgCl_2$ and glass beads and cellular debris pelleted at low speed (10 min, 4000 rpm). The supernatant was then cleared by ultracentrifugation (50.1 Ti, 30 min, 15000 rpm). 4 ml of the cleared lysate were incubated with 160 µl Protein G-dynabeads (Life Technologies) (50% slurry, preincubated with 40 µl anti-Pol II antibody (8WG16, Millipore)) for 4h at 4°C. Beads were repeatedly washed with TNG + 1.5mM $MgCl_2$ and eluted with 250 µl elution buffer (50 mM Tris-HCl, pH 7.5, 10 mM EDTA, 1% SDS) for 10 min at 65°C. The RNA contained in the elution fraction was phenol extracted, DNase-treated and analysed by RT-PCR.

SUPPLEMENTAL TABLES

Table S1. Overlap between genes in the Mmi1 Regulon and Mmi1 CRAC Data, Related to Figure 1.

**Gene** (Chen et al., 2011)	**Systematic Name**	**Mmi1 CRAC**	**Comment**
mug8	SPAC32A11.01	yes	
ssm4	SPAC27D7.13c	no	
mug10	SPAC57A10.04	yes	
mug9	SPCC70.09c	yes	
mcp7	SPAC13A11.03	yes	
rec8	SPBC29A10.14	yes	
mei4	SPBC32H8.11	yes	
-	SPBC1921.04c	Anti-sense only	
ubi4	SPBC337.08c	yes	
crs1	SPBC2G2.09c	yes	
arp1	SPBC1347.12	yes	
-	SPBC19F8.04c	Anti-sense only	
meu32	SPAP27G11.08c	Anti-sense only	
mug1	SPCC11E10.03	no	Immediately downstream of Mmi1-regulated gene SPCC11E10.01
dic1	SPBC646.17c	yes	
mug45	SPBP8B7.04	yes	
tht2	SPAC23C4.07	yes	
-	SPAC6C3.05	yes	
meu1	SPAC1556.06	no	
mug45	SPCC1393.07c	yes	
spo5	SPBC29A10.02	yes	
mcp5	SPBC216.02	yes	
dil1	SPAC458.04c	no	
rep1	SPBC2D10.06	yes	
rec25	SPAC17A5.18c	no	
mcp6	SPBC582.06c	yes	
rec11	SPCC4E9.01c	no	
rec10	SPAC25G10.04c	no	Immediately downstream of Mmi1-bound gene his1
rec27	SPBC577.05c	no	
sme2	sme2	yes	

Table S2. Comparison of CRAC data with polyA+ RNA-seq data, Related to Figure 2. – see xlsx file

Table S3. Analyses of *mmi1Δ* and wt RNA-seq data, Related to Figure 2. – see xlsx file

Table S4. Protein-coding genes with intronic Mmi1 CRAC cDNA reads, sorted by read number, Related to Figure 3.

**Gene**	**Systematic Name**
rps2202	SPAC5D6.01
dbp2	SPBP8B7.16c
mpg1	SPCC1906.01
oga1	SPBC16A3.08c
cdc13	SPBC582.03
SPBC660.16	SPBC660.16
cpc2	SPAC6B12.15
cts1	SPAC10F6.03c
rad24	SPAC8E11.02c
SPBC19G7.17	SPBC19G7.17
pub3	SPBC16E9.11c
rpp0	SPCC18.14c
rpl3801	SPBC577.02
tpz1	SPAC6F6.16c
rlp7	SPAC664.06
SPAC1687.17c	SPAC1687.17c
SPBC1734.10c	SPBC1734.10c
SPBP8B7.15c	SPBP8B7.15c
SPCC1795.07	SPCC1795.07
SPCC830.09c	SPCC830.09c
atg12	SPAC1783.06c
hob1	SPBC21D10.12
mak10	SPBC1861.03
mvp1	SPAC3A11.06

Table S5. Oligonucleotides used in this study, Related to Experimental Procedures. – see xlsx file

Table S6. Yeast strains used in this study, Related to Experimental Procedures. – see xlsx file

SUPPLEMENTAL FIGURES

Kilchert et al., Figure S1

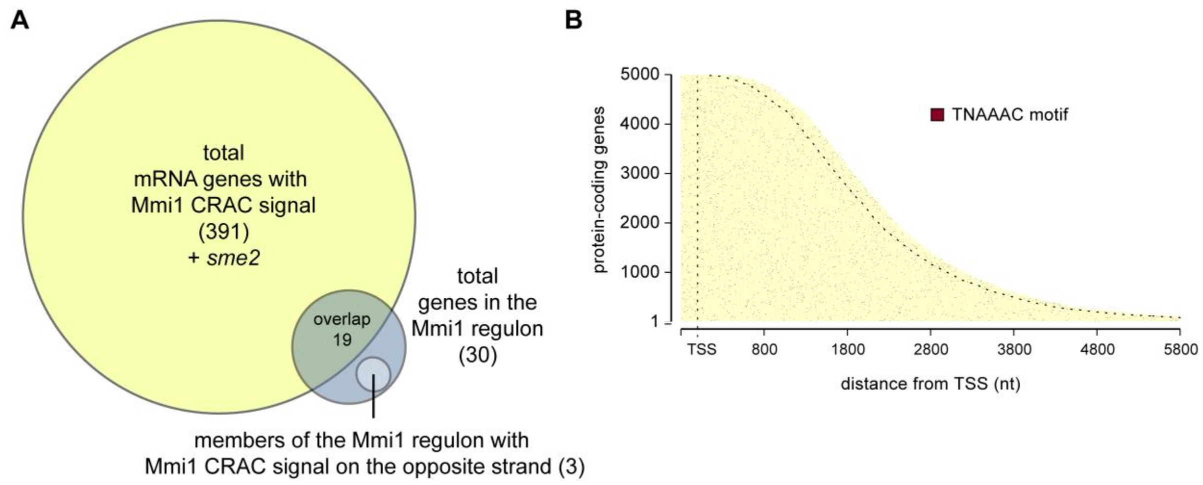

Figure S1. Mmi1 CRAC analysis. Related to Figure 1.

(**A**) Venn diagram showing the overlap between the 391 mRNAs cross-linked to Mmi1 in the CRAC experiments and the 30 members of the "Mmi1 regulon" (Chen et al., 2011). One ncRNA (*sme2*) had been assigned to the regulon and was also included in this graph. See also Table S1. (**B**) Heat map of TNAAAC motif distribution in protein-coding genes. Genes were sorted according to length and aligned at the transcription start site (TSS). The region shown includes 200 bp up- and downstream of each annotated transcript (pink). The dotted lines indicate the position of the annotated TSS and termination site. TNAAAC motifs are indicated in brown.

Kilchert et al., Figure S2

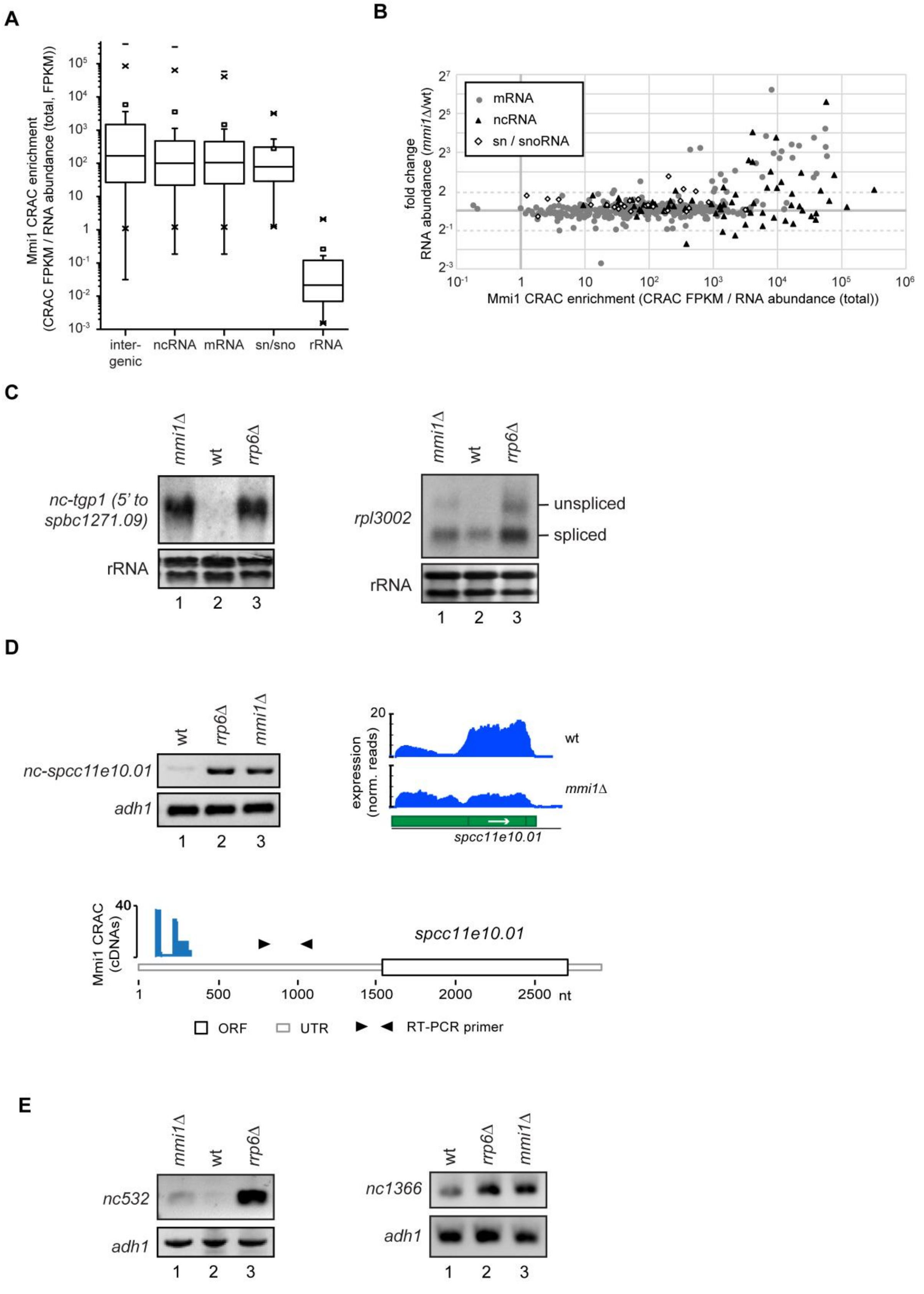

Figure S2. Validation of Mmi1 targets identified by CRAC. Related to Figure 2.

(**A**) Box plot of Mmi1 CRAC enrichment of different transcript classes. Boxes represent 25-75%, whiskers 1.5-fold interquartile range. The maximum value (-), median line (–), mean (▫), and 1-99% range (x) are indicated. (**B**) Correlation between Mmi1 CRAC and expression fold change (RNA-seq). Circles denote mRNAs, triangles ncRNAs, and diamonds sn/snoRNAs. Open and filled markers denote pA- and pA+ transcripts, respectively. (**C**) *nc-tgp1* and *rpl3002* Northern analysis of RNA extracted from the indicated strains. (**D**) *spcc11e10.01* was proposed to be regulated by an upstream ncRNA (Lee et al., 2013). Similar to *pho1* or *tgp1*, we find the ncRNA to be a target of Mmi1. RT-PCR for the ncRNA (position of oligos indicated below), RNA-seq analysis and CRAC cDNA reads over the locus are shown. (**E**) RT-PCR analysis of *nc532* and *nc1366* on total RNA extracted from the indicated strains.

Kilchert et al., Figure S3

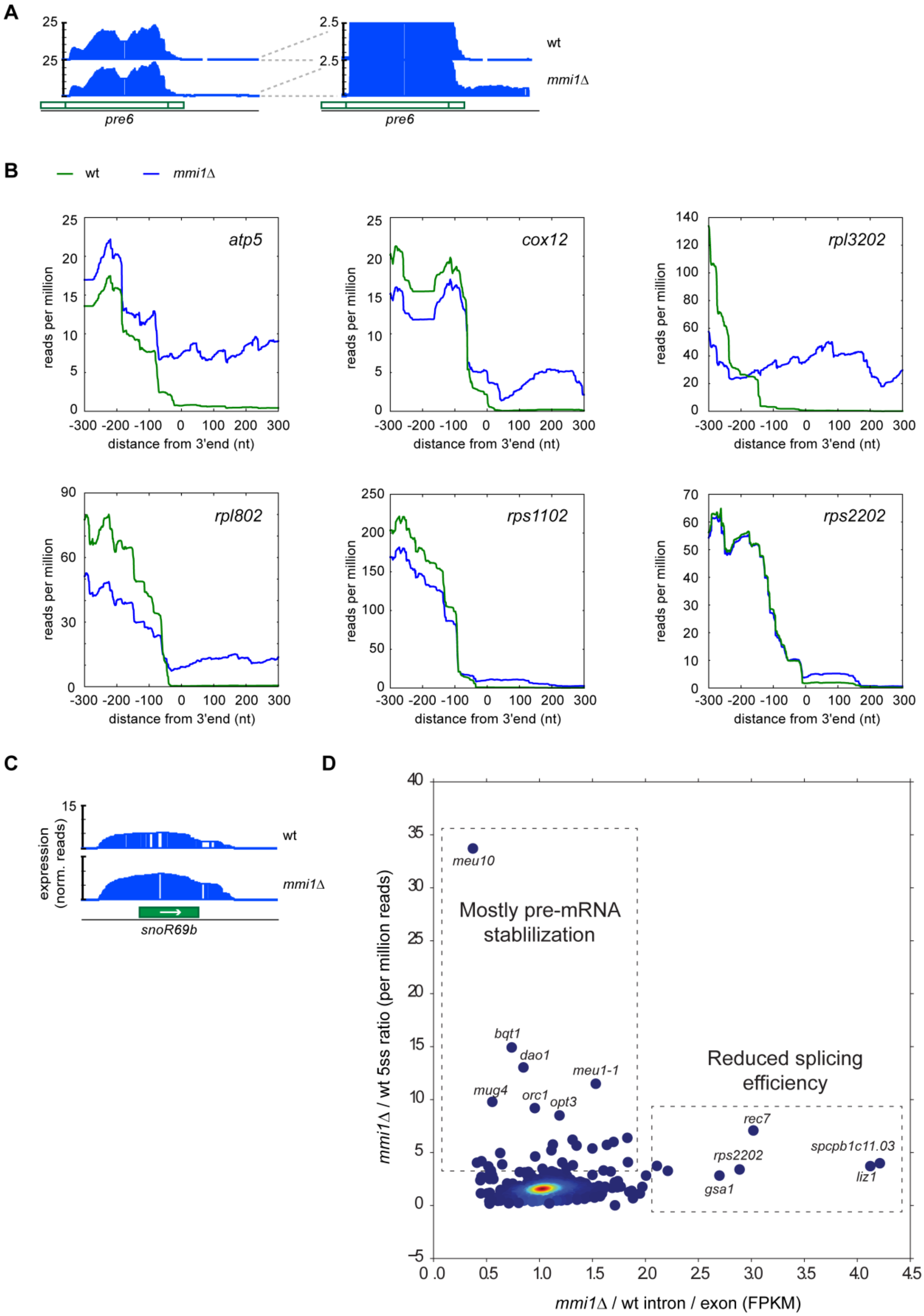

Figure S3. Analysis of gene features differentially regulated in *mmiΔ*. Related to Figure 2.

(**A**) RNA-seq analysis of gene with increased 3'extended reads in *mmi1Δ*. On the right side, the scale is increased ten-fold to resolve the 3'extended species. (**B**) RNA-seq traces of genes likely to have a termination defect in *mmi1Δ*. (**C**) RNA-seq analysis of *snoR69b*. (**D**) Effects of *mmi1Δ* on intron-containing genes were evaluated by plotting the ratio of 5'ss exon-intron boundary reads found in *mmi1Δ* and wt (normalized to per million mapped reads) against the change of the intron/exon FPKM ratio for each transcript.

Kilchert et al., Figure S4

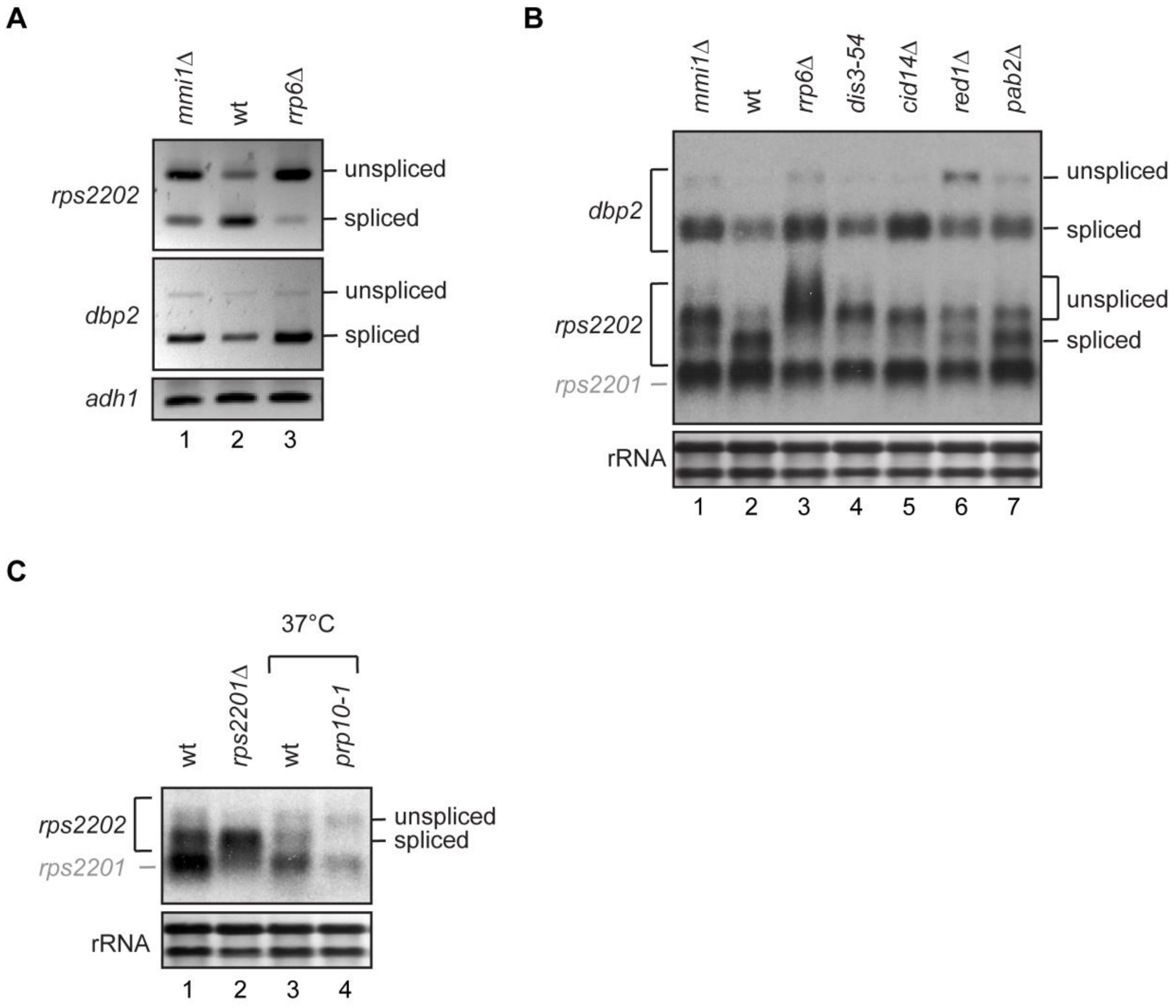

Figure S4. Mmi1 binding to introns regulates the accumulation of spliced product. Related to Figure 3.

(**A**) RT-PCR analysis of *rps2202* and *dbp2* on total RNA extracted from the indicated strains using intron-spanning primers. Levels of *adh1* are shown as control. (**B**) Northern analysis of *rps2202* and *dbp2* on total RNA extracted from the indicated strains. (**C**) Northern analysis of *rps2202* on total RNA extracted from the indicated strains. Because of high sequence conservation, the probe also detects the *rps2201* paralogue. This cross-reactive band disappears when *rps2201* is deleted (lane 2). The splicing mutant *prp10-1* and a wt were grown in YES at 25°C, then shifted to 37°C for 2h prior to RNA extraction. The band corresponding to spliced *rps2202* is strongly reduced in the splicing mutant (lane 4).

Kilchert et al., Figure S5

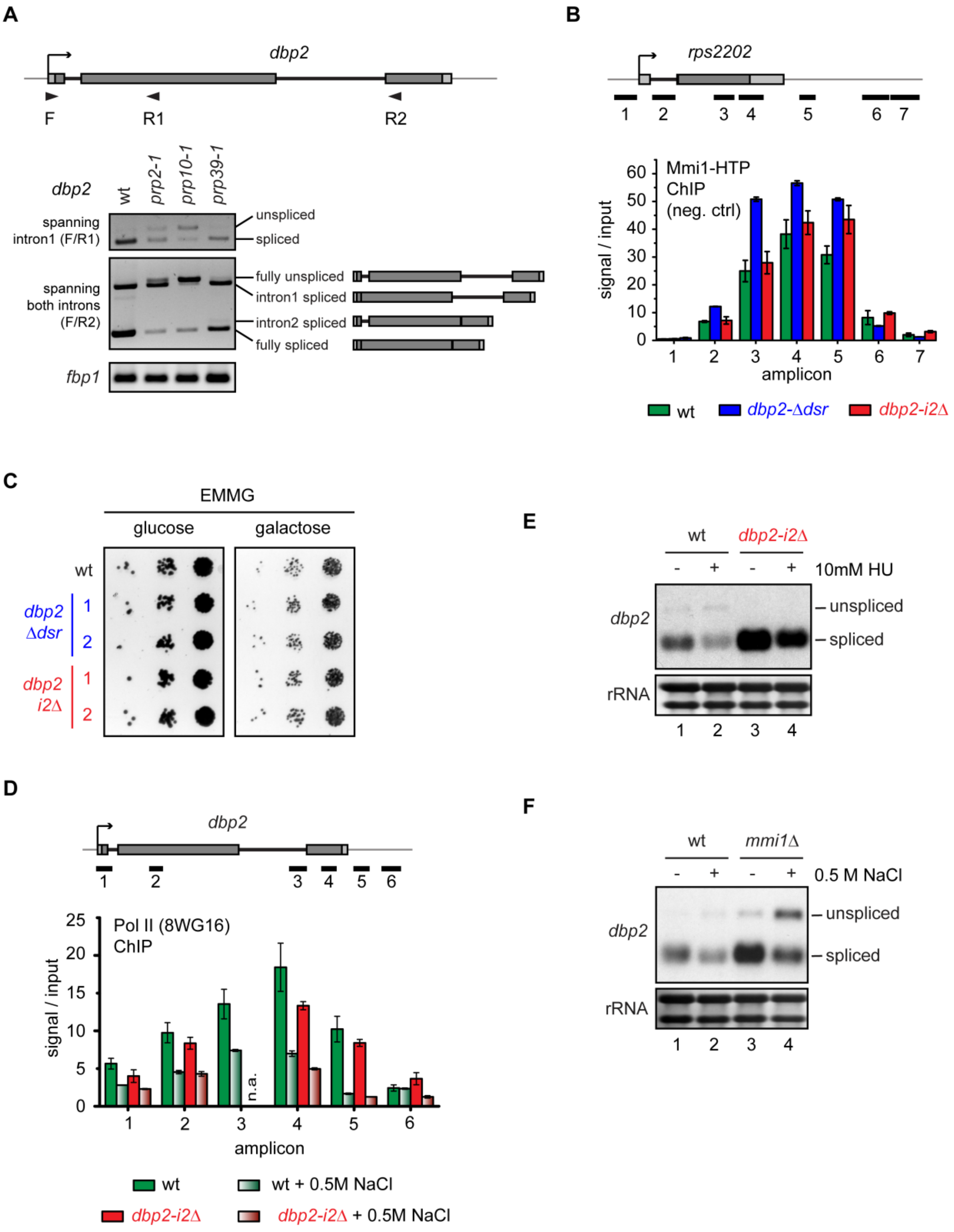

Figure S5. *dbp2* intron 2 is involved in negative expression regulation. Related to Figure 4.

(**A**) RT-PCR analysis of *dbp2* on total RNA extracted from wt and three splicing mutants (*prp2-1*, *prp10-1* and *prp39-1*) grown in YES at 25°C, then shifted to 37°C for 2h using two different sets of

intron-spanning primers. The amplicons are indicated on the right of the gel using oligonucleotides F, R1 and R2.. Levels of *fbp1* are shown as control. (**B**) ChIP analysis of Mmi1-HTP recruitment across the *rps2202* locus as a negative control. Positions of the amplicons are indicated above the bar plot. Error bars represent SEM of at least two biological replicates. (**C**) Test for growth on different carbon sources. wt, *dbp2-Δdsr* or *dbp2-i2Δ* (two clones for each mutant) were grown to log phase in YES at 30°C, then serially diluted onto EMMG plates containing either glucose or galactose/raffinose as a carbon source. The plates were then incubated at 30°C and photographed. (**D**) ChIP analysis of Pol II recruitment across the *dbp2* locus using a Pol II antibody (8WG16). Chromatin was extracted from the indicated strains grown in YES with or without added 0.5M NaCl for at least 24h at 30°C. Positions of the amplicons are indicated above the bar plot. Error bars represent standard error of the mean (SEM) of at least two biological replicates. (**E**) Northern analysis of *dbp2* on total RNA extracted from the indicated strains grown in YES with or without addition of 10 mM hydroxyurea (HU) for 3h at 30°C. (**F**) Northern analysis of *dbp2* on total RNA extracted from the indicated strains grown in YES with or without added NaCl for at least 24h at 30°C. Note that for the +NaCl samples, 10 µg total RNA instead of 8 µg were loaded per lane.

Kilchert et al., Figure S6

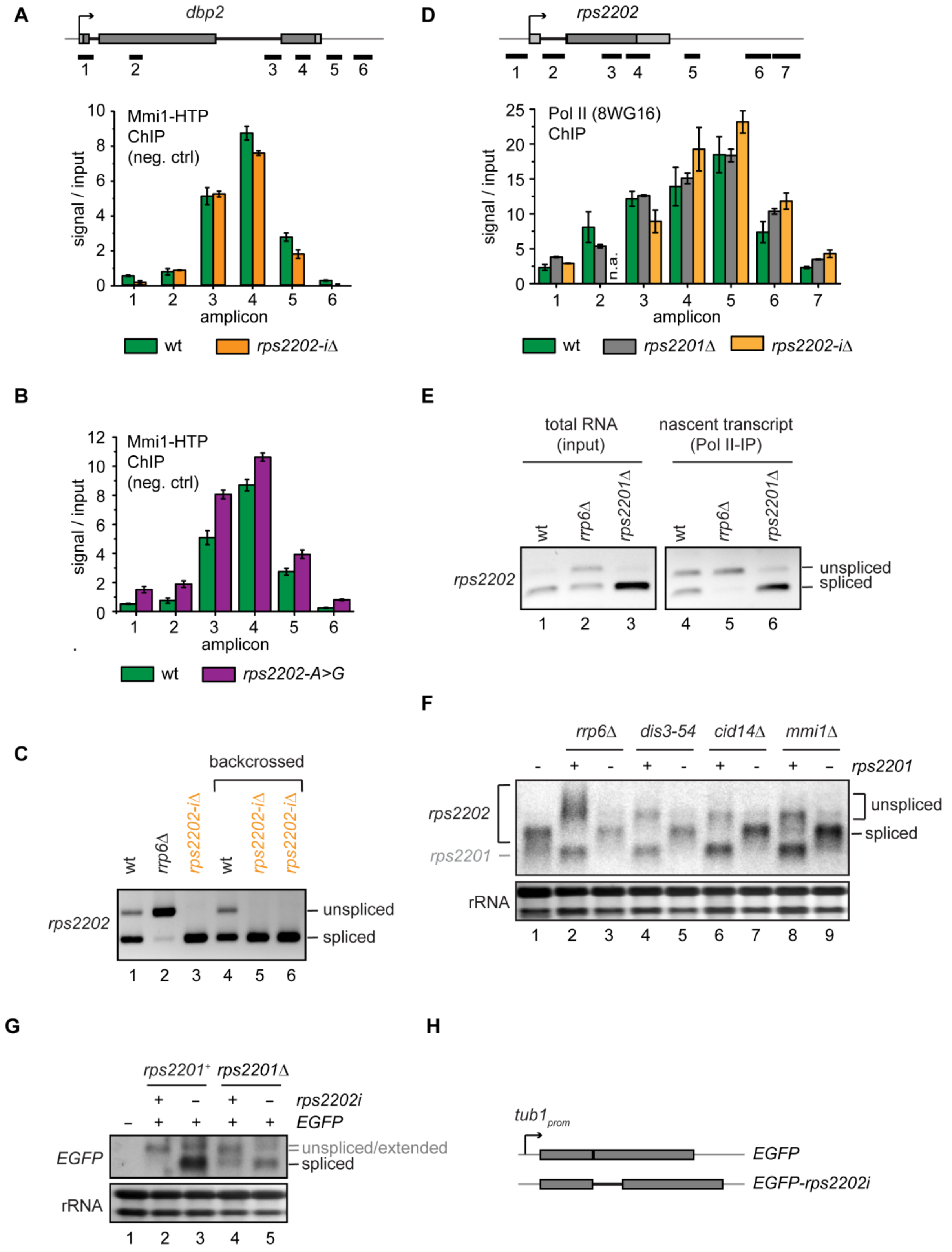

Figure S6. Recruitment of Mmi1 to *rps2202* intron occurs in a context of paralogue-dependent negative splicing regulation. Related to Figure 5.

(**A** and **B**) ChIP analysis of Mmi1-HTP recruitment across the *dbp2* locus as a negative control. Positions of the amplicons are indicated above the bar plot. Error bars represent SEM of at least two biological replicates. (**C**) RT-PCR analysis of *rps2202* on total RNA extracted from the indicated strains. The *rps2202-iΔ* mutant was backcrossed into wt background to verify that the phenotype co-segregated with the mutation (lanes 3-5). (**D)** ChIP analysis of Pol II recruitment across the *rps2202* locus using a Pol II antibody (8WG16). Positions of the amplicons are indicated above the bar plot. Error bars represent SEM of at least two biological replicates. (**E**) Splicing of nascent *rps2202* transcript in different mutants evaluated by analysis of Pol II-associated transcripts. Cells were grown in YES at 30°C, lysates prepared and Pol II IP-ed with 8WG16 antibody. RNA extracted from input and IP samples was analysed by RT-PCR. (**F**) Northern analysis of *rps2202* on total RNA extracted from *rps2201Δ* and nuclear surveillance mutants *rrp6Δ, dis3-54*, *cid14Δ* or *mmi1Δ* with or without additional deletion of *rps2201*. Bands corresponding to spliced and unspliced *rps2202* transcripts and the paralogue *rps2201* are indicated. (**G**) Northern analysis of EGFP on total RNA extracted from wt or *rps2201Δ* with a *EGFP* reporter driven by the *tub1* promoter integrated into the *leu1* locus. The reporter gene was either intron-free or contained the *rps2202* introns, as indicated. Note that levels of the unspliced construct are difficult to assess because it co-migrates with an extended species of the reporter transcript. (**H**) Schematics of the constructs used in (G).

SUPPLEMENTAL REFERENCES

Bähler, J., Wu, J.Q., Longtine, M.S., Shah, N.G., McKenzie, A., Steever, A.B., Wach, A., Philippsen, P., and Pringle, J.R. (1998). Heterologous modules for efficient and versatile PCR-based gene targeting in Schizosaccharomyces pombe. Yeast *14*, 943–951.

Bayne, E.H., Portoso, M., Kagansky, A., Kos-Braun, I.C., Urano, T., Ekwall, K., Alves, F., Rappsilber, J., and Allshire, R.C. (2008). Splicing factors facilitate RNAi-directed silencing in fission yeast. Science *322*, 602–606.

Bühler, M., Spies, N., Bartel, D.P., and Moazed, D. (2008). TRAMP-mediated RNA surveillance prevents spurious entry of RNAs into the Schizosaccharomyces pombe siRNA pathway. Nat. Struct. Mol. Biol. *15*, 1015–1023.

Gao, J., Kan, F., Wagnon, J.L., Storey, A.J., Protacio, R.U., Davidson, M.K., and Wahls, W.P. (2014). Rapid, efficient and precise allele replacement in the fission yeast Schizosaccharomyces pombe. Curr. Genet. *60*, 109–119.

Matsuyama, A., Shirai, A., Yashiroda, Y., Kamata, A., Horinouchi, S., and Yoshida, M. (2004). pDUAL, a multipurpose, multicopy vector capable of chromosomal integration in fission yeast. Yeast *21*, 1289–1305.

Nicolas, E., Yamada, T., Cam, H.P., Fitzgerald, P.C., Kobayashi, R., and Grewal, S.I.S. (2007). Distinct roles of HDAC complexes in promoter silencing, antisense suppression and DNA damage protection. Nat. Struct. Mol. Biol. *14*, 372–380.

Ohkura, H., Adachi, Y., Kinoshita, N., Niwa, O., Toda, T., and Yanagida, M. (1988). Cold-sensitive and caffeine-supersensitive mutants of the Schizosaccharomyces pombe dis genes implicated in sister chromatid separation during mitosis. EMBO J. *7*, 1465–1473.
